# Exploring Relevant Features for EEG-Based Investigation of Sound Perception in Naturalistic Soundscapes

**DOI:** 10.1523/ENEURO.0287-24.2024

**Published:** 2025-01-16

**Authors:** Thorge Haupt, Marc Rosenkranz, Martin G. Bleichner

**Affiliations:** ^1^Neurophysiology of Everyday Life Group, Department of Psychology, Carl von Ossietzky Universität Oldenburg, Oldenburg 26129, Germany; ^2^Research Center for Neurosensory Science, Carl von Ossietzky Universität Oldenburg, Oldenburg 26129, Germany

**Keywords:** acoustic representations, electroencephalography (EEG), naturalistic sound perception, neural encoding

## Abstract

A comprehensive analysis of everyday sound perception can be achieved using electroencephalography (EEG) with the concurrent acquisition of information about the environment. While extensive research has been dedicated to speech perception, the complexities of auditory perception within everyday environments, specifically the types of information and the key features to extract, remain less explored. Our study aims to systematically investigate the relevance of different feature categories: discrete sound-identity markers, general cognitive state information, and acoustic representations, including discrete sound onset, the envelope, and mel-spectrogram. Using continuous data analysis, we contrast different features in terms of their predictive power for unseen data and thus their distinct contributions to explaining neural data. For this, we analyze data from a complex audio-visual motor task using a naturalistic soundscape. The results demonstrated that the feature sets that explain the most neural variability were a combination of highly detailed acoustic features with a comprehensive description of specific sound onsets. Furthermore, it showed that established features can be applied to complex soundscapes. Crucially, the outcome hinged on excluding periods devoid of sound onsets in the analysis in the case of the discrete features. Our study highlights the importance to comprehensively describe the soundscape, using acoustic and non-acoustic aspects, to fully understand the dynamics of sound perception in complex situations. This approach can serve as a foundation for future studies aiming to investigate sound perception in natural settings.

## Significance Statement

This study is an important step in our broader research endeavor, which aims to understand sound perception in everyday life. Although conducted in a stationary setting, this research provides foundational insights into necessary environmental information to obtain to understand concurrent neural responses. We delved into the analysis of various acoustic features, sound-identity labeling, and cognitive information, with the goal of refining neural models related to sound perception. Our findings particularly highlight the need for a thorough analysis and description of complex soundscapes. Our study provides key considerations for future research in sound perception across various contexts, from laboratory settings to mobile EEG technologies, and paves the way for investigations into more naturalistic environments, advancing the field of auditory neuroscience.

## Introduction

Mobile electroencephalography (EEG) has opened new avenues for studying neural activity beyond the lab (BTL), offering insights to expand our current understanding of brain function in real-world settings (Gramann et al., 2011; Studnicki et al., 2022; Hölle and Bleichner, 2023). One critical aspect of interpreting EEG data recorded BTL is understanding the environmental context driving the neural response (Holdgraf et al., 2017; Robbins et al., 2021). Thus, sufficient information about the environment need to be captured to accurately determine how it influences neural responses. For BTL recordings, however, there is typically a tradeoff between the aspects that can be captured and the overall mobility implicating unobtrusive recordings (Bateson et al., 2017). Therefore, a selective approach is required, focusing on environmental aspects that are within the scope of the setup and are most pertinent to the research objectives (Bleichner and Debener, 2017).

In the study of auditory perception, particularly in understanding how we perceive naturally occurring sounds in everyday life, it has yet to be shown, which auditory features are most relevant to understand the concurrent neural response.

**Figure 1.**
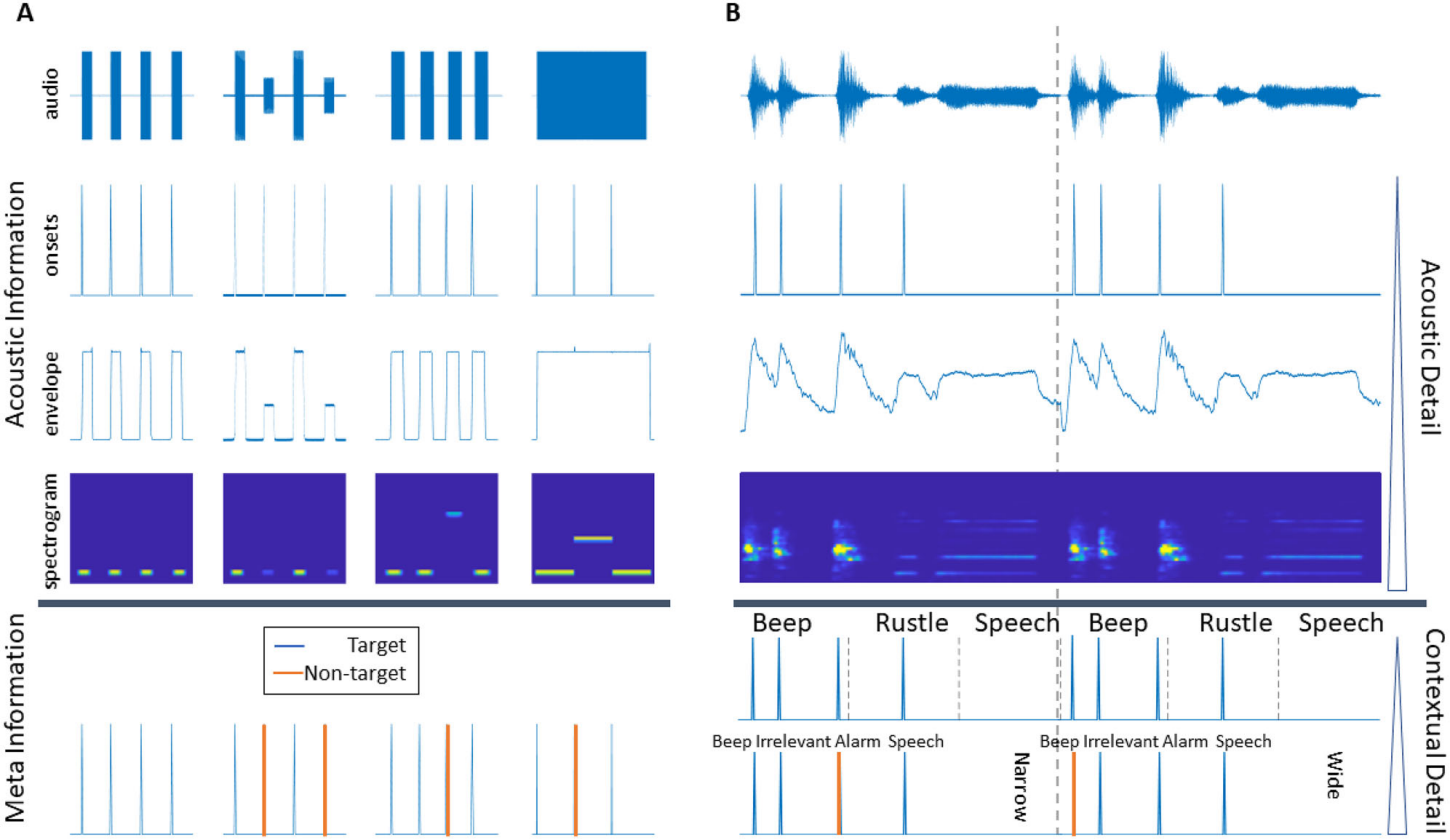
***A***, This panel illustrates the levels of abstraction with which acoustic features depict different tone sequences. Furthermore, it highlights the impact of the acoustic detail the acoustic onset, envelope, and mel-spectrogram capture. In the first tone sequence, onsets reveal the timing of sound occurrence, while the envelope extends this information by showing both the timing and duration of the sound; the mel-spectrogram further adds details about the frequency content. For the second tone sequence, the limitation of onsets becomes apparent, as they do not convey any information about amplitude changes. In the third sequence, the frequency changes are distinctly and exclusively captured by the mel-spectrogram, with the onset and envelope methods remaining indifferent to these frequency variations. The fourth sequence underscores a similar limitation of the envelope, which fails to distinguish frequency variations in a continuous tone. The lower section of this panel demonstrates the crucial role of meta-information. For instance, understanding which sound in an experiment requires a behavioral response cannot be discerned from the acoustic features alone. Meta-information thus complements the acoustic analysis by providing essential contextual and functional information. ***B***, This panel showcases acoustic feature representations—onsets, envelope, and mel spectrum—for a complex naturalistic soundscape. Additionally, it highlights the diversity of meta-information that can describe these soundscapes. The upper row features continuous classification from Yamnet, providing labels for individual sound segments. The lower row presents manual annotations, categorizing sounds into different conditions (narrow and wide) and distinct types (beep, irrelevant, alarm, and speech sounds). These labels, informed by task-specific knowledge, offer deeper contextual and descriptive insights into the soundscape, illustrating the multi-layered nature of acoustic analysis in naturalistic settings.

One well-studied feature category depicts the acoustic properties of the soundscape on different levels of abstraction (de Heer et al., 2017; Hamilton et al., 2021). Starting from the most abstract, discrete sound onsets, to continuous broadband amplitude changes captured by the envelope, to the mel-spectrogram depicting power fluctuations across different frequency bands over time (Fig. 1*A*). However, the choice of abstraction is crucial; with greater abstraction relevant fine structure information about the audio is lost.

Beyond the acoustic depiction, additional layers of information (meta-information), as illustrated in Figure 1*B*, should be considered for a comprehensive understanding of auditory processing (Robbins et al., 2021). For instance, Zuk et al. (2020) have demonstrated that the EEG response to music and speech stimuli is stronger compared to other natural sounds. Here, knowing the sound identity (SI), i.e., the category of a sound, such as speech, rustle, etc., is a valuable insight for neural model building (Fig. 1*B*). Beyond the soundscape description, the cognitive state, or “cognitive priors,” (CPs) of the participant, such as how attentional resources are allocated, profoundly affect how auditory information is processed and, consequently, the resulting EEG responses (Obleser and Kayser, 2019; Holtze et al., 2021; Rosenkranz et al., 2023).

From a pragmatic perspective, the effort required to acquire various auditory features can vary significantly. Where Acoustic features can be directly and readily obtained from the auditory waveform, deriving meta-information presents a greater challenge. These cannot be directly extracted from the audio signal but require additional processes (Robbins et al., 2021).

The question, then, is which features are relevant to the construction of neural models, particularly in the context of naturalistic soundscapes. The current study aims to explore the optimal feature selection and the impact of including SI and CPs in neural models, bearing in mind the practical limitations of data collection in natural settings.

Our approach employs continuous forward modeling to investigate the link between the derived features and their representation in the neural signal (Ding and Simon, 2012; Crosse et al., 2016; Holdgraf et al., 2017; Hamilton and Huth, 2020). This methodology allows for a nuanced understanding of the neural underpinnings of auditory perception and examine our two primary study goals: First, if SI and CP information enhance the estimation of neural response models. This involves examining whether meta-information provides a significant advantage in modeling the neural correlates of auditory perception. Second, we seek to understand the extent to which the level of acoustic detail influences the effectiveness of neural response model estimation for natural soundscapes. This aspect of the study focuses on assessing how varying degrees of detail in acoustic feature representation impact the accuracy and robustness of our neural models.

By addressing these two objectives, our research contributes to a deeper understanding of how different types of auditory information are encoded in neural responses, particularly in rich, real-world acoustic environments.

## Method

### Data set

#### Experimental design

The analysis in this study is based on an existing dataset by Rosenkranz et al. (2023). The study aimed to investigate how altering attentional focus affects the perception of auditory information. This was done in two separate conditions, where participants had to respond to two different tones while they engaged in a complex audio-visual motor task. These tones are integrated within a soundscape designed to mimic a surgical auditory environment.

Specifically, the complex task involved an adapted 3-dimensional version of a Tetris game, where participants received occasional auditory instructions to place a specific Tetris piece at a distinct location. The auditory background soundscape consisted of various sounds, including alarms, monitor beeps, and speech as they are common in an operation room. Additionally, a nonrelevant coherent conversation split into smaller segments was played to simulate background conversation.

In each of the conditions, the participants listened to the same audio track. Only the instruction regarding the specific tone participants had to respond to changed. In the first condition, participants were instructed to press the space bar when hearing an alarm sound, which introduced a narrow focus. In the second condition, participants had to respond to beep tones which were played from multiple directions together with other sounds, necessitating a more comprehensive (wide) attentional focus. For both conditions, the task-relevant instructions had to be monitored concurrently. In this study, we refer to conditional differences as CP, as these have been shown to induce two different brain states. For a detailed procedural account, please refer to the open source material of the previous study: Rosenkranz et al. (2023).

#### Auditory stimuli

The soundscape included three added tones, each of which was played 48 times. A hospital alarm and a task-irrelevant hospital monitor sound, which each lasted approximately 200 ms. The last sound that was included, a beep tone, was generated using Matlab, with a frequency of 800 Hz, and a duration of 60 ms.

Participants heard 12 times one of four instructions they had to comply with (“Place the next stone in the [upper left—lower left—upper right—lower right] corner”). The same instruction was never played consecutively. The irrelevant speech segments were taken from podcast conversations unrelated to the task and medical setting. There were in total 48 task-irrelevant speech segments that were presented in a pre-defined order and only once. Each snippet lasted approximately 3.5 ( ± 1.5) s and was extracted using Audacity®.

All the extracted sounds were processed in Matlab, such that their root-mean-squared (RMS) value matched that of the average RMS of all sounds. Adjusting for differences in loudness was achieved by applying specific gain parameters to each auditory sound. Additionally, the tones were spatially separated using the head-related impulse function (Kayser et al., 2009).

#### Code accessibility

The code described in the paper is freely available online at https://github.com/ThorgeHaupt/RelevantFeaturesSoundPerception. The analyzed dataset can be found under https://zenodo.org/records/7147701. A Dell Precision 3,650 Tower running Microsoft Windows 10 Education was used.

10.1523/ENEURO.0287-24.2024.d1 Extended Data 1 Download Extended Data 1, ZIP file.

#### Data acquisition

For measuring the EEG, participants were fitted with 24 Ag/AgCI passive electrodes according to the 10–20 international system (EasyCap GmbH, Hersching Germany). The collected data was amplified using a wireless SMARTING system (mBrainTrain, Belgrade, Serbia) and referenced to Fz, and grounded to AFz. The data was sampled at 500 Hz and the impedance of electrodes was kept below 20 Ω before the recording. The audio presented during the experiment was sampled at 44.1 kHz. All data streams recorded were synchronized using the Lab Recorder software, which is based on the Lab Streaming Layer. Before measuring EEG data, participants were informed about the procedure and had to sign the informed consent.

#### Preprocessing of EEG data

The EEG data were preprocessed in MATLAB (version 2021a, MathWorks, Natick, MA) using the EEGlab plugin and custom scripts. For detecting artifacts in the data we ran an Independent Component Analysis (ICA). To obtain optimal ICA weights, Winkler et al. (2015) proposed a preprocessing pipeline that runs separately from the actual preprocessing of the data that will be analyzed later on. In other words, the preprocessing of the data for the ICA analysis does not impact the data used for analysis later on, as merely the ICA weights are extracted and added back to the raw data. In particular, we merged the two experimental conditions for each participant. Subsequently, the data have been resampled to 250 Hz, after which a high- and then a low-pass filter were applied (*pop_firws*(EEG, “fcutoff,” 1,“ftype,” “highpass,” “wtype,” “hann,” “forder,” 568), *pop_firws*(…,“fcutoff,” 42,“ftype,” “lowpass,”…,“forder,” 128)). The cut-off frequencies were chosen to eliminate drifts and line-noise to obtain optimal ICA weight estimates (Winkler et al., 2015). Furthermore, channels with poor signal quality were identified and removed using the *clean_channels* function. Lastly, the data were segmented into 1-second epochs and converted to double digits. Artifactual epochs were removed using the *pop_jointprob* function using a threshold of 3 standard deviations. The ICA was computed using the *pop_runica* function utilizing the extended version. The weights were added back to the raw and unfiltered data of each condition. Next, the ICA components were automatically flagged as either, muscle, eye, heart, line noise, or channel noise using the *pop_icaflag* function within a corresponding, specified threshold range of probabilities ([0.7 1;0.7 1;0.6 1;0.7 1;0.7 1]).

Following artifact removal, the raw data were filtered using the same filter setup as previously described, with slight modifications to the filter order and cut-off frequencies. First, the low-pass filter was applied: fcut 20 Hz, forder: 100. The data were subsequently resampled to 100 Hz and subjected to high-pass filtering: fcutoff 0.3 Hz, forder 518. The choice of lower filter order for the low pass filter was motivated by recommendations of Crosse et al. (2021), as to minimize artifacts caused by sharp roll-over introduced by higher-order filters. Reducing the passband to [0.3 20] Hz was done according to literature from speech tracking, demonstrating that the most auditory processing activity is found in the lower frequency ranges (Di Liberto et al., 2015; Crosse et al., 2016). Finally, the data was referenced to the mastoids (TP9/TP10).

### Sound features

A full description of the soundscape is available, including acoustic information, the label of specific sounds (SI), behavioral responses, and conditional information. Here, we derived three types of features: acoustic features (AC), SI markers, and CP.

**Figure 2.**
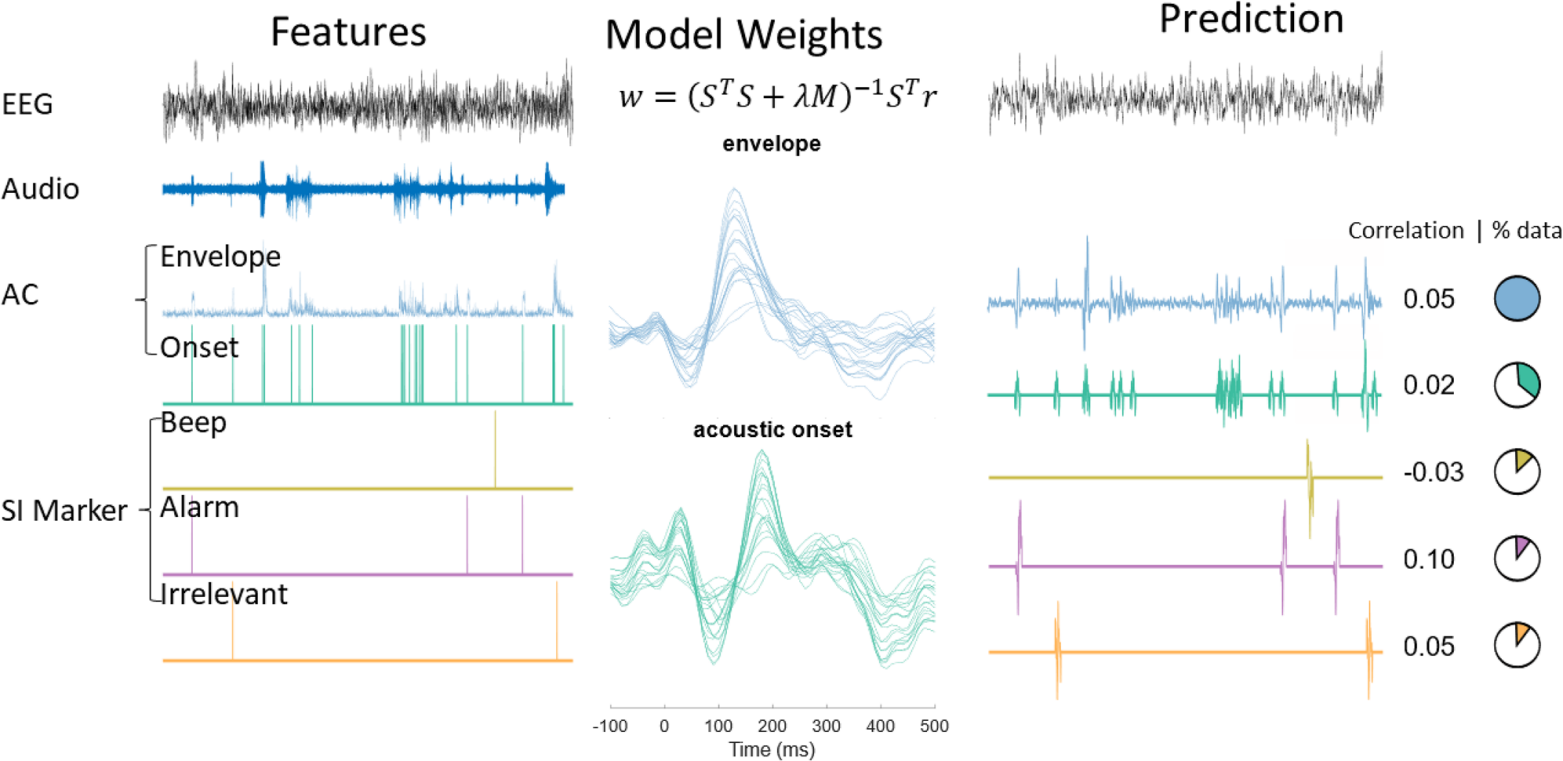
This figure depicts the process of forward modeling. From the audio several features are extracted. Using the EEG data and the features the temporal response function is derived for each EEG channel. This temporal response function is then used to predict unseen neural data based on the corresponding feature information. The resulting prediction is correlated with the actual signal. If discrete features are used, then not every sample can be predicted. This is visualized in the pie charts, showing the proportion of samples that can be predicted. AC = acoustic features, SI = sound identity.

The acoustic features that we derived in our study were acoustic onsets (i.e., transient sound events), the envelope, and the mel spectrum. These features vary in their level of acoustic detail, with acoustic onsets being the most sparse and the mel spectrogram providing the most detailed representation. Importantly, acoustic onsets are discrete in contrast to the continuous envelope and mel-spectrogram. This has crucial consequences for predicting unseen data, as for sufficiently spaced onsets, not every sample can be predicted (Fig. 2). These features have been validated in many different studies using speech stimuli (de Heer et al., 2017; Brodbeck et al., 2018; Daube et al., 2019; Desai et al., 2021; Mesik and Wojtczak, 2023). Here we want to test whether these features can be extended to soundscapes with non-speech soundscapes.

SI and CP were available through the meta-information. This information is not necessarily readily available from everyday recordings (Hölle and Bleichner, 2023). For the current study, we selected a subset of tones for which SI markers and CP information were present; alarm, beep, and irrelevant tone. The experimental relevance of the first two tones changed between conditions, whereas the last tone remained irrelevant throughout the experiment.

In addition to directly comparing different features, we also explored multi-feature models. For instance, we combined several acoustic features’ information to assess whether their properties were processed differently in the brain. A combination of acoustic onsets and the envelope yielded a model we referred to as OnsEnv. Alternatively, we investigated the effect of providing SI and CP information in addition to acoustic features. Here, combining acoustic onsets with alarm tones yielded a model we referred to as AlarmOns. This approach proved particularly valuable for assessing overall model improvement when incorporating additional information and examining the overlap and unique contributions of each feature. Throughout the analyses, we employed the combination of different features multiple times.

It is important to note that when combining features, it was advised to normalize them to a common scale. This was crucial as the magnitude of the feature impacted the derivation of the model weights (Crosse et al., 2021). Thus, when different features were combined, we applied a min–max normalization, such that the values were bound to be between [0 1]. We opted for this particular normalization as it transformed each non-binary feature to share the same scale as that of the acoustic onsets and SI marker.

#### Acoustic onsets

Acoustic onsets were derived for the entire soundscape, indicating the time point when the intensity of the soundscape exceeded a predefined threshold. The acoustic onsets here were represented as binary vector, where 1 indicates sound onset and 0 no sound onset. As we are interested in the impact acoustic detail has on explaining neural variability, we opted for the most parsimonious representation of the soundscape: the onset of a sound. The extraction of onsets occurred unsupervised, thus for any sound, indiscriminate of experimental relevance or the presence of meta-information. To derive the onsets, we used an energy novelty function (Müller, 2021). Initially, the audio waveform was squared and smoothed using a Hann window (length = 2,048 and overlap = 128 samples). The resulting smoothed signal was then logarithmically compressed with a gamma scaling factor of 10 to approximate the perception of sound intensities in humans.

Subsequently, the difference function was applied to obtain the rate of change in sound intensity, which was further smoothed. The resulting approximation of the first-order derivative was half-rectified to produce the final output of the energy novelty function. This smoothed rate of change in sound intensity served as a representation of sound gain.

To identify acoustic onsets, we employed a threshold peak detection function. In this function, a peak was detected when the sound gain exceeded the threshold value. The resulting feature vector was binary, where a value of one indicated the presence of a sound onset.

#### Envelope

A plethora of studies have shown that EEG-measured neural activity tracks the acoustic envelope of auditory stimuli (Luo and Poeppel, 2007; Giraud and Poeppel, 2012; Drennan and Lalor, 2019), even with transparent setups (Mirkovic et al., 2016; Holtze et al., 2022). There are different ways to derive the acoustic envelope (Petersen et al., 2017; Oganian and Chang, 2019). To highlight that not only the feature but also the way of deriving the envelope impact model estimates, we have used two different ways. For all envelope computations, we first converted the stereo audio recording to mono and computed the envelope over the entire signal.

The first method, as described by Petersen et al. (2017), involves taking the absolute value of the Hilbert transform of the audio. To ensure a smooth envelope, a third-order Butterworth low-pass filter with a cut-off frequency of 30 Hz was applied to the resulting signal. Finally, the envelope was downsampled to match the sampling rate of the EEG signal.

Second, the mTRFenvelope function from the mTRF toolbox was used. This method involved computing the signal’s power, followed by resampling through a moving average filter. To compress the signal, the square root of the power was applied, using a compression parameter of $\log_{10}\;(2)$.

The resulting envelopes are highly correlated rho = 0.94 indicating that they contain almost the same information content. As can be seen from Extended Data Figure 4-1, the spectrum differences occur mostly in the below 1 Hz spectrum and do not follow any systematicity. The stark deviation around 0 Hz is caused by an offset. Given the high correlation and almost non-existent frequency differences, we inspected the estimated model weights. Here, edge artefacts for the envelope are visible at extreme latency values. Reducing the time lags manually and thus removing the edge artefacts did not impact the model performance.

In conclusion, albeit being very similar, the mTRF contains more power in the lower frequency ranges, which is highly relevant for tracking of soundscapes (Howard and Poeppel, 2010; Deoisres et al., 2023). We believe that this may be the reason why the mTRF envelope outperformed the envelope. Henceforth, only the results of the mTRF envelope are considered.

#### Mel-spectrogram

The mel-spectrogram is a time-frequency decomposition of a continuous signal. The 40 frequency bins are spaced according to the mel-scale, which has been shown to mimic the auditory perception of the human ear (Stevens et al., 1937). We have used the MIR-toolbox (Lartillot et al., 2008) function with the following settings: *mirenvlope(“audio.wav,” “Spectro,” “Mel,” “Sampling,” 100)*. The output was adjusted to the length of the EEG signal.

The mel-spectrogram offers the most acoustic detail out of the three acoustic features selected. While the broadband envelope depicts amplitude changes over time, the mel-spectrogram represents power changes in the different frequency bins over time. Like the envelope, the mel-spectrogram describes the soundscape at every sample.

#### SI markers

Part of the original experiment was the presentation of three sounds that were embedded in the soundscape. Through available meta-information, we extracted their marked onset and experimental condition information. We embedded this information in a feature vector, for each tone and condition respectively, in the form of ones and zeros. Here, ones mark the onset of the tone, whereas every other sample is 0. This is in contrast to the acoustic features, which solely comprise acoustic data and lack information pertaining to the SI and conditional information.

Similar to the acoustic onsets, however, SI markers are discrete. Thus, using these features we cannot make predictions regarding unseen data at every sample point. Due to the sound specificity of the features, we can predict even fewer samples compared to the acoustic onsets. This yielded a tradeoff between building highly sound specific models and a proportion of explainable data (Fig. 2).

#### Cognitive priors

Besides acoustic characteristics, the cognitive state of the perceiver critically impacts the neural response to any type of auditory stimulation. For example, a CP could be the instruction given to a participant to pay attention to a specific sound. When a participant is instructed to focus on a particular sound, the neural response is generally larger compared to when the sound is unattended (Rosenkranz et al., 2023). This information cannot be derived from acoustic properties alone but is crucial for understanding the neural response.

In the current data set, there were two conditions where participants receive separate instruction: attend to the alarm sound (narrow), or attend to the beep tone (wide). The attentional manipulation should impact the perception of the soundscape and thus the underlying neural activity. Including this information in the model estimation should lead to more neural variability being explained. Specifically, we have integrated this information by building models separately for the different conditions. Exemplary for the alarm tone, we used two feature vectors, one indicating the sound onset in the narrow condition and another vector depicting the alarm tone onsets in the wide condition.

### mTRF

To analyze the neural time series, we employed the mTRF toolbox developed by Crosse et al. (2016) in MATLAB. This toolbox enables one to derive a set of weights, which establish the relationship between the output of a system to a given set of input vectors through convolution. In our study, the output of the system is the instantaneous neural time series denoted as *r*(*t*, *c*), where *c* = 1, …, *C* represents the channel index and *t* = 1, …, *T* the time points. The measured time series can be modeled as the convolution of a set of channel *c*-specific weights *w* at a distinct timelag *τ* with the stimulus features shifted by *τ*. This approach aims to replicate the brain’s processing, wherein the response to a stimulus is not immediate but rather delayed by an unknown duration. The activity not accounted for by the response weights is captured by the residual term ε(t,c).

$$r(t,c)=\sum_{\tau }\omega (\tau ,c)s(t-\tau )+\varepsilon (t,c).$$

In this study, each of the features’ set of channel weights was used to interpret the morphology and topography, investigate model performance, compute multivariate models, and utilized for cross-prediction.

The determination of the Temporal Response Function (TRF) involves solving an optimization problem aimed at minimizing the Mean Squared Error (MSE) between the actual and predicted neural time series:

$$\min_{\epsilon (t,n)}=\sum_{t}[r(t,c)-\hat{r}(t,c){]}^{2}.$$

The solution is obtained by computing the weight vector **w**:

$$w=(S^{T}S{)}^{-1}S^{T}r,$$

where S is the design matrix containing the stimulus time series at the different time lags *τ*. The dimensionality of the resulting design matrix is sample points by numbers of features and lags (T*N^*ft*^*τ*). In this study, this would yield weight dimensionality of 40 × 61 × 22 for the mel-spectrogram and 1 × 61 × 22 for all other AC and SI features. Furthermore, the stimulus matrix is padded with zeros at non-zeros lags to ensure causality (Mesgarani et al., 2009). The matrix operation where the transposed matrix **S** is multiplied by the neural time series **r** yields an inner product between the stimulus and neural time series at each time lag *τ*, indicating the similarity between stimulus and neural data. The autocorrelation of the stimulus is accounted for by the inverse of the autocovariance (note: the inverse operation can be read as a division for square matrices). The resulting weight matrix **w** is of dimensionality *N*^*ft*^ x *τ* x *C* and describes the set of weights that optimally predict the neural time-series at channel *C*.

Generally, a time lag of [−100 500] ms is used, unless stated otherwise. Furthermore, if cross-validation is applied, the lambda parameter search is conducted on a linearly spaced set of values ranging from 10e−4 to 10e4 in steps of 10.

Now that the mathematical operations underlying the analysis of continuous data have been defined, the data set described, and the derivation of features explained, we will detail the analyses that compare the different features.

### Analyses

The data analysis was performed using MATLAB (version R2021a, MathWorks, Natick, MA) with the aid of custom scripts. Part of the analyses were inspired by the work of Desai et al. (2021). For all of the following analyses, we split the data into 6 segments (*M* = 3.14 min, SD = 0.18), where each consists of the neural data and the concurrent soundscape. At least one segment always served as a held-out test set for which the performance metric was computed. The remaining segments were used for deriving model weights and cross-validation parameter estimation. The neural data was split accordingly and z-scored to ensure comparability between segments.

The model testing is done by predicting the neural response based on the held-out test set. The performance metric used to assess each feature was the correlation between the predicted and actual neural time series, providing a measure of the model’s ability to capture the underlying neural activity. We opted to contrast the different correlational distributions using the Wilcoxon sign rank tests at *α* = 0.05, since the assumption of normality was violated for several prediction distributions. We have refrained from computing the corresponding effect size, as the interpretation of such does not provide meaningful information regarding the magnitude of differences between prediction performance. Multiple testing correction was applied according to Benjamini and Yekutieli (2001) FDR method. For all analyses, the chance level of the correlation for using acoustic onsets was estimated by creating a random acoustic onset vector. For this, we randomly shuffled the temporal position of the acoustic onsets, while preserving the inter-onset interval of the original data. This was done for each condition and participant 100 times and the resulting 95% confidence interval was determined as noise floor.

#### Nested model design

The first crucial step in our analyses was to establish whether the selected meta-information provides meaningful information for model estimation beyond the existing acoustic depictions. We opted for a nested design, where different factors of meta-information were iteratively added to model estimation and resulting prediction values compared. In particular, we tested three levels. The acoustic features represented the base level onto which we added sound identity, and at last, integrated CP information.

The first level involved testing the different acoustic features and contrasting their model prediction accuracy. For this, we concatenated the separate EEG data sets and corresponding feature vectors. These were partitioned into 10 segments through which iterated such that each segment became a test set once. In each iteration, the regularization parameter was determined on the remaining nine segments, and model weights estimated. In the last step, we predicted the neural data on the held-out test set and averaged the resulting correlation values over channels. As this was done for each iteration, we finally averaged over iterations.

To address the impact of adding SI information to model estimation we added the SI marker of the three tones to each of the acoustic features separately, i.e., combining the acoustic onsets once with the alarm, the irrelevant, or the beep tone. The same training and testing procedure as described earlier was repeated.

The last step involved taking CPs into consideration. To assess their impact, we split each of the tones from the prior analysis (i.e., alarm, irrelevant, and beep) into two separate feature vectors. One feature vector indicated the occurrence of the tone in the narrow condition and the other feature vector indicated the occurrence of the tone in the wide condition. These vectors were zero-padded such that the length remained consistent with that of the neural data.

The statistical analysis involved contrasting the different levels of information content for each of the acoustic features and paired SI markers. The corresponding correlational distributions were compared using the Wilcoxon-signed rank test and multiple comparison corrections was applied as outlined above. As an additional analysis, we extracted the model weights corresponding to either condition for each tone and compared these using cluster-based permutation testing as detailed in Mirkovic et al. (2019) using the fieldtrip toolbox (Oostenveld et al., 2011). This was done to test whether the different CPs from the study would impact model weight estimation.

#### Variance-partitioning

To address in how far the different models explained similar neural activity, we used variance partitioning (de Heer et al., 2017; Crosse et al., 2021; Desai et al., 2021). This analysis allowed us to investigate the unique variance explained of each feature and to pinpoint the degree to which combining different features led to model improvement.

The underlying concept of variance partitioning is that if models A and B share some degree of similarity, the explained variance of the combined model should be less than the sum of the individual models (A+B>A∪B). In other words, the features are not independent. In our study, the correlational values are transformed into the variance explained *R*^2^.

When determining the variance explained from each single and combined model, the unique and mutual explained variance can be computed as A∩B=A+B−A∪B. This can be extended to three variables as well, where:

A∩B∩C=A∪B∪C+A+B+C−A∪B−B∪C−A∪C.

In the case of the current study, an example would be to derive a set of model weights based on the envelope, onset, and mel-spectrogram information. For this particular example, we could test how overall model performance improves compared to single feature models i.e., $A_{ons}\cup B_{env}\cup C_{mel}vs.[A_{ons},B_{env},C_{mel}]$, but also which feature contributed the most to the improvement and which features are redundant.

$$A_{ons}/(B_{env}\cup C_{mel})=A_{ons}-A_{ons}\cup B_{env}-A_{ons}\cup C_{mel}.$$

We segmented the data into six segments, five for training and cross-validation, and one held out test set for which we calculated the *R*^2^. This was done for all features and their combinations. For the subsequent variance partitioning analysis, we considered only models with a maximum of three different features.

In our study, we calculated the *R*^2^ value for each feature directly from the average channel correlational values. However, in some cases where we sought to determine the unique contribution of a feature in a multi-feature model, the resulting *R*^2^ value was negative. The negative *R*^2^ values, theoretically impossible, according to set theory, are likely caused by overfitting of noise and by too large predictor sets. To address this issue, we introduced a bias estimator *post hoc* based on de Heer et al. (2017) work. The optimal bias estimator was derived based on the underlying constraint that variance partitioning should yield values that are at least zero. Here we expect that model similarity is expressed by the degree of overlap.

#### Cross-prediction

The similarity of information content between each model was assessed by the variance partitioning analysis. Our next goal was to evaluate the generalizability of a model to predict the neural response to different features. Concerning the results of the variance partitioning analysis, we expected the shared variance observed to reflect the strength of the correlation in this analysis.

In detail, we derived model weights on the training set containing information of feature *A* and feature *B*. Then we used the derived weights (*A*_*w*_,*B*_*w*_) to predict neural time series on a test set based on feature *B* information. An example of such a procedure was to select acoustic onsets and mTRF envelope for model training, and applying both of the derived weights on the mTRF envelope of the test data set to predict the neural time series. We pre-defined pairs of features to be tested, which were derived for each participant and condition.

To ensure robust conclusions regarding cross-prediction scores, each of the segments (N = 6) served as a testing set once. Within each fold, we derived two sets of model weights, corresponding to either of the feature pair. These weights were used to estimate the neural time series using both the original feature information and the information from the other feature. This approach yielded four prediction scores (averaged over channels) for each segment: two within-prediction scores and two cross-prediction scores. The resulting cross-validation correlational scores were averaged over folds. The mel-spectrogram was excluded from this analysis, as feature dimensions had to be consistent.

We adopted a comparative approach by analyzing the relationships between within and cross-prediction scores for the different feature pairs. The selected approach eliminated the need for normalizing explained variance, as some features inherently explain more variance than others, thus rendering a comparison based on absolute prediction scores unsuitable.

A high correlation between scores suggests that if the within-feature model weights accurately predict the neural time-series, so do the model weights from another feature. This indicates the generalizability of the model weights to other feature information.

For some feature pairs, weights looked highly similar in their trajectory, but peaks were shifted in time. This led to low cross-prediction scores, despite model similarity. Correcting for that shift improved scores tremendously. Further testing revealed that the weights’ difference did not imply separate neural processes, but was caused by the feature’s properties. This further warrants caution when interpreting model weights (Haufe et al., 2014; Popov et al., 2018; Kriegeskorte and Douglas, 2019).

## Results

The main purpose of this study was to investigate feasibility of different features to explain neural data in the context of a naturalistic soundscape. Specifically, we compared acoustic features with respect to their level of acoustic detail. Furthermore, the additional benefit of including meta-information, specifically sound identity markers and CPs, in the neural model estimation was assessed systematically.

### Nested model analysis

In the first analysis, we investigated the impact of including meta-information in the model weight estimation. Specifically, we wanted to test whether adding information of SI markers and CPs would enhance model estimation. Here we combined each of the three different acoustic features with SI markers separately. This was done to determine the impact of sound identity information on model estimation.

**Figure 3.**
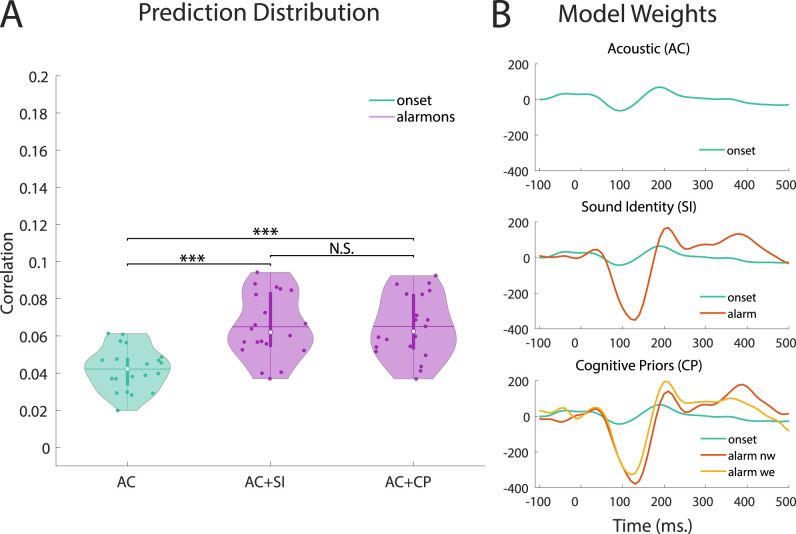
Exemplary model building of combining acoustic onset information with alarm tones and the respective CPs. ***A***, Statistical comparison of prediction distributions based on AC = Acoustic, combined with either SI = sound identity, CP = cognitive prior information. *p < 0.05, **p < 0.01, ***p < 0.000, N.S. = non-significant ***B***, Example of model weights for acoustic onsets alone, extended by SI, or CP information. A comparison of the condition effect to the results of Rosenkranz et al. (2023) can be found in the Extended Data Figure 3-1. The *x*-axis displays the time lags for the different weights. In the last plot, the nw = narrow and we = wide condition weights are displayed for the alarm tone.

When combining the acoustic features with any of the three sound identity markers, model performance improved compared to the acoustic features alone. Combining the mTRF envelope with the alarm tone (*W* = 0.0, *Z* = −3.92, *p* = 0.001), with the irrelevant (*W* = 0.0, *Z* = −3.92, *p* = 0.001), and with the beep tone (*W* = 1, *Z* = −3.88, *p* = 0.001) significantly improved model performance. Similarly, for the acoustic onsets, specifying experimental tones in the form of the alarm tone (*W* = 0.0 *Z* = −3.92, *p* = 0.001) (Fig. 3), with the irrelevant (*W* = 0.0 *Z* = −3.92, *p* = 0.001) and with the beep tone (*W* = 1, *Z* = −3.88, *p* = 0.001) improved model prediction compared to using only the acoustic onsets. The mel-spectrogram also improved, but by less compared to the other two acoustic features. Here specifying the alarm tone (*W* = 12, *Z* = −3.47, *p* = 0.0036), the irrelevant (*W* = 0, *Z* = −3.92, *p* = 0.001) and the beep tone (*W* = 32, *Z* = −2.73, *p* = 0.034) improved model prediction.

10.1523/ENEURO.0287-24.2024.f3-1 Figure 3-1 Upper panel shows the TRF weights of the alarm tone for the two conditions. The grey shaded area marks the window of interest as reported by Rosenkranz et al. (2023). The black lines are the clusters detected by the permutation testing with the corresponding topographies. The lower panel shows the same but for the beep tone. Download Figure 3-1, TIF file.

Next, we tested whether additional consideration of CPs would yield further model improvement. Contrary to the expectations, including CP information did not improve any feature combination, but led to decreased performance for the irrelevant and beep tone in combination with mTRF envelope (Irr: *W* = 198, *Z* = 3.47, *p* = 0.0036; Beep: *W* = 184, *Z* = 2.94, *p* = 0.018), and the acoustic onsets with the irrelevant tone (*W* = 195, *Z* = 3.36, *p* = 0.005).

The models that included CP information outperformed the respective acoustic models in predicting unseen data. The only case where this effect was not observed, was the combination of the mel spectrogram and CP information of the beep tone (*W* = 40, *Z* = 2.43, *p* = 0.073).

In order to validate the finding that CP did not yield any beneficial information for model estimation, we applied cluster-based permutation testing on the derived model weights of the sound identity markers. The results revealed no significant cluster that compared to the windows selected in Rosenkranz et al. (2023), which found significant condition differences for both the alarm [336–432] ms. and beep [308–404] ms. tone. Since we applied a more conservative measure of permutation testing compared to the original linear mixed-model analysis for the pre-specified windows, we re-ran our analysis using the same statistical testing reported in Rosenkranz et al. (2023). Again, the results did not yield any significant differences between conditions (alarm: ß =0.1564, *SE* = 0.1444, *t*(19) = 1.083, *p* = 0.29; beep: ß =0.1759, *SE* = 0.1839, *t*(19) = 0.975, *p* = 0.35). A visualization in (Extended Data Fig. 3-1) revealed that the TRFs of the narrow and wide condition did not deviate strongly during the pre-defined period, for neither the alarm nor the beep tone marker, explaining the non-significant findings. The TRF trajectories deviate from those of Rosenkranz et al. (2023), which can be attributed to different preprocessing decisions. Albeit no significant differences were found in the pre-defined windows, significant deviations were detected for other latencies (alarm:[180 310], [360 800] ms, beep: [400 800] ms.). However, when testing whether these differences in TRF weights would impact the prediction accuracy, no significant differences were found, thereby supporting our previous finding of the nested model analysis. Based on this result, we do not consider condition differences to be relevant in the interpretation of the results of the subsequent analyses.

### Variance partitioning

One shortcoming of the previous analysis was that it did not reveal whether any of the derived models explained similar aspects of the underlying neural processes. Thus, the current analysis aimed to determine the degree of similarity of the different models. First, we computed the model prediction accuracies of the different features and used these values for the subsequent computation of the variance partitioning. The statistical assessment was performed on the model predictions only.

**Figure 4.**
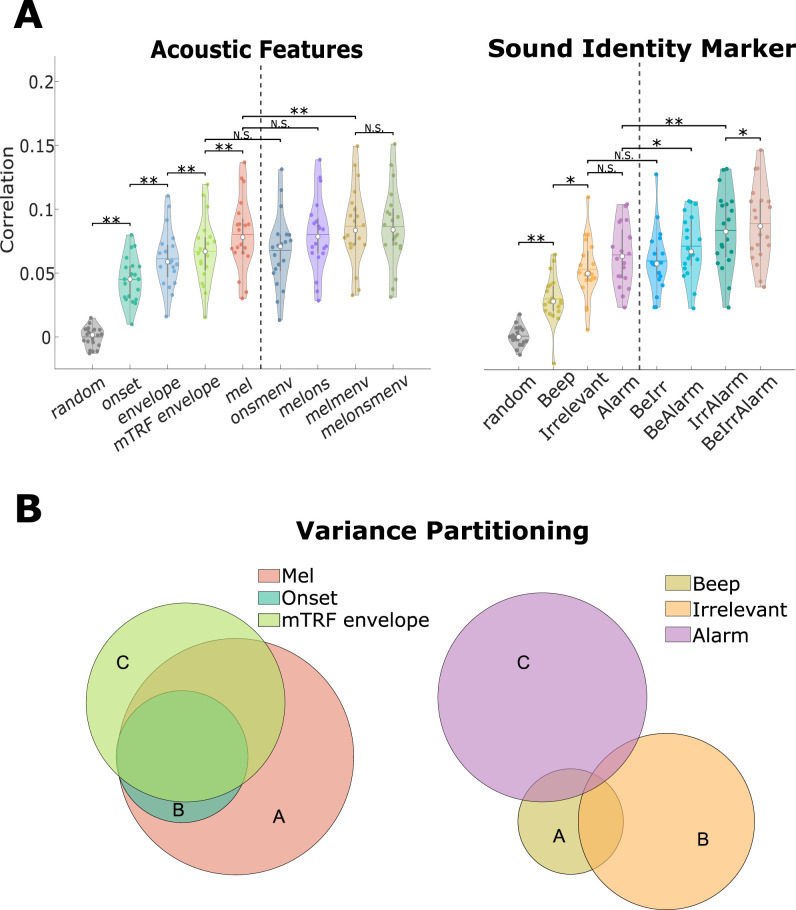
***A***, A comparison of the prediction distributions of the acoustic features and their combinations. *p < 0.05, **p < 0.01, ***p < 0.000, N.S. = non-significant. A detailed comparison between the envelope and mTRF envelope can be found in Extended Data Figure 4-1. ***B***, The result of the variance partitioning showing the unique contribution and shared explained data. The letters correspond to Table 1, which displays the values of the variance partitioning analysis.

The results of contrasting the acoustic features showed that model prediction differed between them. A direct comparison between models revealed that the mel-spectrogram yielded model weights that were most successful in predicting unseen neural data compared to all other base models (mel—ons: *W* 210, *Z* = 3.92, *p* = 0.002; mel—menv: *W* = 207, *Z* = 3.36, *p* = 0.010). The model based on the acoustic onsets performed significantly worse than the other models. Furthermore, the comparison between the two different envelope models showed that the mTRF-implemented function to derive the envelope yielded a better performance (*W* = 210, *Z* = 3.92, *p* = 0.002). Each model’s performance presented in Figure 4*A* was above chance level.

10.1523/ENEURO.0287-24.2024.f4-1 Figure 4-1 The left panel shows the distribution of the prediction accuracy for the envelope and mTRF envelope model. Significance is tested at * p < 0.05, **p < 0.01, ***p < 0.000. The panel in the middle shows the model weights averaged over participants, conditions, and channels. The panel on the right highlights the frequency decomposition of the difference curve of the mTRF envelope and the envelope. Download Figure 4-1, TIF file.

**Table 1.** Shows the variance explained for the acoustic (AC) and sound identity (SI) features

		*r* ^2^	$r_{unq}^{2}$	A ∩B	A ∩C	B ∩C	A ∩B ∩C
	Beep (A)	0.0009	0.0007	0.0003	0.0002	0.0001	0.0003
SI	Irr (B)	0.0028	0.0027				
	Alarm (C)	0.0041	0.0041				
	Mel (A)	0.0072	0.0031	0.0023	0.0039	0.0020	0.0020
AC	Ons (B)	0.0023	0				
	mEnv (C)	0.0052	0.0012				

Specifically, the first two columns display the total and unique variance explained by each feature. The last four columns highlight the shared variance ( ∩), where A refers to the first, B to the second, and C to the third feature, for SI and AC respectively (Fig. 4).

Comparing the results of combining the acoustic features pairwise revealed that performance did not differ when acoustic onsets were added to either the mTRF envelope (*W* = 85 *Z* = −0.75, *p* = 1) or the mel-spectrogram (*W* = 108, *Z* = 0.11, *p* = 1) compared to respective, better performing, base model. Combining the mTRF envelope with the mel-spectrogram, however, significantly outperformed the respective base (menv: *W* = 3, *Z* = 3.81, *p* = 0.003; mel: *W* = 0, *Z* = 3.92, *p* = 0.002) and any model combination that included the acoustic onsets. Any acoustic base model that was outperformed by the other acoustic base model was also outperformed by their combined model.

These results were also reflected in the variance partitioning. Nearly all of the variance that was explained by the acoustic onsets was also captured by the mel-spectrogram ( $r_{ons}^{2}=0.0023,r_{ovlp}^{2}=0.0023$) (Fig. 4*B*). Similarly, most of the variance explained by the mTRF envelope was contained in the mel-spectrogram ( $r_{ovlp}^{2}=0.0039$). The mTRF envelope did, however, explain unique aspects of the neural processing ( $r_{unq}^{2}=0.0012$) (Table 1).

Similar to the acoustic features each SI marker model yielded model weights that predicted neural data above chance level (Fig. 4*A*). Contrasting the different SI markers with each other, the alarm tone yielded the highest and the beep tone the lowest performance. While the alarm tone’s performance did not differ significantly from that of the irrelevant tone (*W* = 171, *Z* = 2.46, *p* = 0.136), both differed significantly from the beep tone’s prediction accuracy (alarm: *W* = 200, *Z* = 3.55, *p* = 0.005; irrelevant: *W* = 191, *Z* = 3.21, *p* = 0.015).

Inspecting the results of the pairwise combination of SI markers revealed that only the combination of the beep and irrelevant tone did not outperform both base models (Beep: *W* = 207, *Z* = 3.81, *p* = 0.003; Irr: *W* = 173, *Z* = 2.539, *p* = 0.112). When all three sound identity markers were used to derive a combined model all of the pairwise combined models were outperformed significantly (IrrAlarm: *W* = 185, *Z* = 2.99, *p* = 0.031; BeIrr: *W* = 210, *Z* = 3.92, *p* = 0.002; BeAlarm: *W* = 206, *Z* = 3.77, *p* = 0.003).

The results of the variance partitioning of the SI marker showed that most models explain relatively more unique variance than shared variance. This finding is unsurprising, given that the sound identity markers describe different sections of the soundscape. There were, however, differences in terms of the proportional amount of unique variance explained between the different base models in their respective pairings. In particular, the beep tone model shared the most overlap with any other base model proportional to its total variance explained ( $r^{2}=0.0009,r_{unq}^{2}=0.0007$). In contrast, if the irrelevant tone was combined with the alarm tone, then most of the explained variance is unique (Alarm: $r_{unq}^{2}=0.0041$; Irr: $r_{unq}^{2}=0.0027;r_{ovlp}^{2}=0.0001$) (Fig. 4*B*, Table 1).

### Proportion explained

One aspect that has not been considered in the previous analysis, but is vital if one aims to compare different sets of features, is the consideration of the features’ structure. For instance, the acoustic envelope depicts amplitude changes over the entire time course and hence allows to relate the ongoing EEG to the ongoing soundscape at each time point. Whereas discrete features, e.g., sound onsets, only allow to model the neural response around specific time points and do not provide a prediction of the neural signal where no sound onsets occur. Considering the typical lag used in our model computation, which covers 500 milliseconds post-stimulus onset, viable predictions are only feasible for periods immediately following an auditory event. In contrast, during “event-free” periods where no auditory onset event is present, the model’s prediction of EEG activity is effectively zero, rendering these predictions non-informative. To determine the impact this distinction has on model prediction, we re-evaluate the accuracy of discrete features for sections where data can be predicted using the analysis pipeline of the variance partitioning.

**Figure 5.**
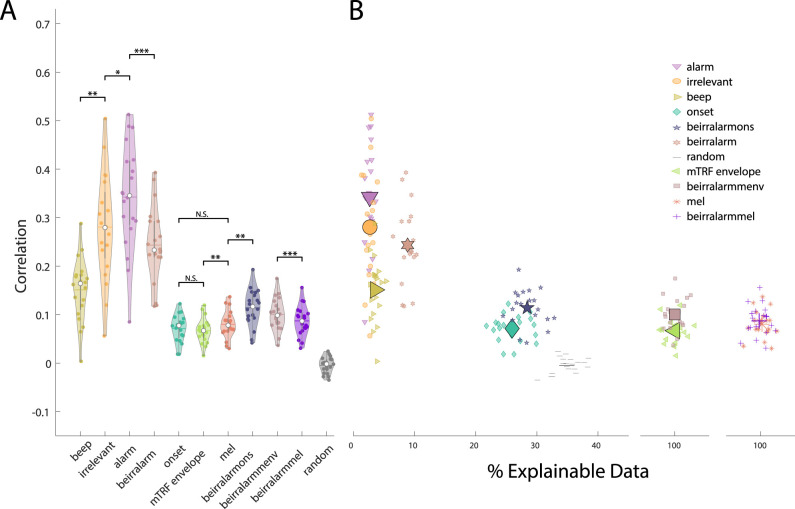
***A***, Displayed are the distributions of prediction accuracies for a subset of different features and feature combinations. Each dot represents the average condition and channel correlation score of one participant. These distributions are the collapsed results over proportion of explainable data as presented in ***B***, The results of the Wilcoxon sign rank test are presented exemplary at *p < 0.05, **p < 0.01***p < 0.000, N.S. = non-significant. ***B***, prediction distributions share the same *y*-axis as that of part A. Additionally, we computed the proportion of data that is explainable using the different features. For the continuous features such as the envelope or mel-spectrogram, the variance was added for visual purposes.

Here, we observed a nonlinear negative trend in terms of prediction accuracy with an increasing proportion of explainable data (Fig. 5). While the sound identity markers contributed only a fraction of the total explainable data (alarm: 3%, irrelevant: 3%, beep: 4%), they showed high prediction accuracies for these small sections. We again observed that the alarm tone yielded the best model predictions (*M* = 0.34, SD = 0.11), followed by the irrelevant (*M* = 0.28, SD = 0.11), and then the beep tone (*M* = 0.15, SD = 0.06). Interestingly, combining these three markers into one model yielded overall more data that can be explained (9%), but with reduced accuracy compared to the separate models (*M* = 0.24, SD = 0.08). The prediction distribution of the combined model appeared to be a linear combination of the results of the three separate models.

Using the acoustic onsets, we could make predictions for roughly 26% of the data using the described TRF settings. For the mTRF envelope and the mel-spectrogram on the other hand, we were able to predict the neural data at every sample. Contrasting the prediction distributions by accounting for the difference in samples where predictions were made, we found that the difference between the acoustic onsets and the mTRF envelope was no longer significant (*W* = 144, *Z* = 1.46, *p* = 0.668). Furthermore, the direct comparison between the acoustic onsets and the mel-spectrogram revealed they were also no longer statistically different (*W* = 155, *Z* = 1.867, *p* = 0.290). The contrast between the mel-spectogram and mTRF envelope remained significant (*W* 0 195, *Z* = 3.36, *p* = 0.004).

The prediction accuracy as well as the proportion of explainable data were improved if the SI markers were added to the acoustic onsets (29%, *M* = 0.11, *SD* = 0.04) (Fig. 5). The increased proportion of data that could be explained indicates that our detection of onsets can be further improved, as seemingly not all SI markers were detected. We believe that the improved prediction accuracy for the acoustic onset and SI markers was not only due to the increased data quantity that could be explained. Rather, we found that the additional information of the SI markers also improved the data prediction qualitatively, as highlighted by finding increased the performance of combining SI markers with the mTRF envelope (*M* = 0.1, SD = 0.03) or mel-spectrogram (*M* = 0.9, SD= 0.03). Since the mTRF envelope and mel-spectrogram made predictions at every sample, the improved prediction accuracy can be attributed to the additional information of the SI markers. Specifically, the combination of SI markers with the mTRF envelope (*W* = 210, *Z* = 3.92, *p* = 0.001) or mel-spectrogram (*W* = 207, *Z* = 3.808, *p* = 0.001) outperformed either base model.

**Figure 6.**
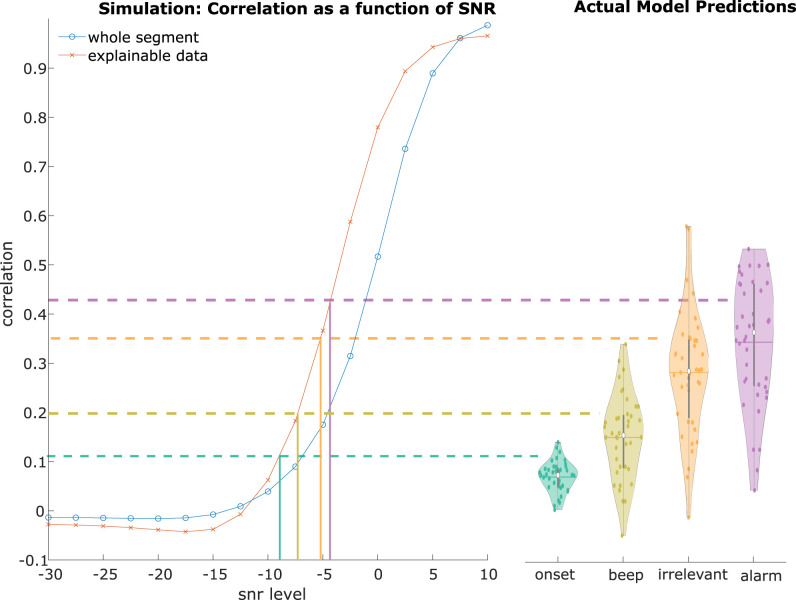
The left panel shows the results of the simulation study. The *x*-axis depicts the different simulated SNR levels in dB. The *y*-axis shows the correlational values. The two graphs show the mean correlational values as a function of SNR levels for either the whole segment (blue) or only where data was predicted (red). The vertical lines show the estimated SNR level for the onset, beep, irrelevant, and alarm tone respectively. The panel on the right shows the prediction scores of the actual data.

The correlational values found for the SI markers for the explainable data periods exceed those commonly found in other speech tracking studies (Drennan and Lalor, 2019; Gillis et al., 2021; Mesik and Wojtczak, 2023). Here, generally, values ranging around 0.02–0.05 are detected. To investigate if the values we observed are plausible we created 10 min of data where we randomly placed 396 triphasic ERP responses (P1-N1-P2). On top of this signal, we added pink noise, where the noise distribution followed the 1/f distribution. The noise was added at various amplitudes achieving SNRs that varied between −30 and 10 dB. We subjected this simulated data to the same analysis pipeline as for the original data, and computed correlation coefficients for the explainable segments as well as for the whole segment (Fig. 6).

Another crucial aspect was determining the SNR level of our sound identity markers and acoustic onsets model at a single trial level. For this, we computed the standard deviation of a baseline period in each trial [−0.1 −0.01] s. and took the mean absolute value of the corresponding ERP from [0 0.5] s. The ratio of these two values was converted to dB and used as a single trial estimate of SNR. Vertical lines represent the SNR values of the different features, and the plot on the right reflects the actual data predictions. As can be seen, the expected correlational values of the simulation are similar to the ones obtained for the actual data. The results for the simulation are consistently higher than the mean correlation values of the real data. The higher values observed in the simulation study may be due to the assumption of identical neural responses for each trial, which is not realistic. Despite this limitation, we believe that our simulation still demonstrates that these high correlation values are possible.

### Cross prediction

The results of the cross-prediction indicated in how far a model trained on one feature would be able to predict data based on other feature information. A positive correlation of prediction scores indicates that segments that are predicted well by the matching feature model, are also predicted well by the other feature model.

Inspecting the corresponding cross-prediction for the mTRF envelope and onset we observed a highly linear relationship. Using mTRF envelope information, we show a high correlation between prediction values using the mTRF envelope model and acoustic onset model. (*ρ* = 0.76, *p* < 0.001). Conversely, using acoustic onset information we show a high correlation using the acoustic onset model and mTRF envelope. (*ρ* = 0.79, *p* < 0.001) (Table 2).

**Table 2.** Table shows the correlational values for the different cross-prediction pairs

		base		
		AC		SI		
		onset	mTRF envelope	alarm	irrelevant	beep
	onset	1	0.76	0.72	0.81	0.49
	mTRF envelope	0.79	1	0.70	0.69	0.38
Cross	alarm	0.69	0.28	1	0.85	0.51
	irrelevant	0.72	0.44	0.81	1	0.63
	beep	0.48	0.18	0.63	0.72	1

The table should be read from left to top. The feature labels on the left are the models that were trained on these features. The labels at the top are feature information used to predict segments using the model on the left. A visualization of the results can be found in Extended Data Table 2-1 and 2-2. All correlations are significant at *p* < 0.01.

10.1523/ENEURO.0287-24.2024.t2-1 Table 2-1 This figure shows the results of the cross-prediction analysis. On the x-axis are the correlational score of the testing data segment with the prediction based on feature information that the model was initially trained on. On the y-axis are the correlational scores for the same segment and feature information as on the x-axis, but using model weights derived from the depicted feature. Download Table 2-1, TIF file.

10.1523/ENEURO.0287-24.2024.t2-2 Table 2-2 This figure shows the results of the cross-prediction analysis for the sound identity marker. On the x-axis are the correlational scores of the testing data segment with the prediction based on feature information that the model was initially trained on. On the y-axis are the correlational scores for the same segment and feature information as on the x-axis, but using model weights derived from the depicted feature. Download Table 2-2, TIF file.

All model cross-predictions showed a significant linear relationship. Here, the beep tone performed better at predicting the alarm (*ρ* = 0.63, *p* < 0.001) or irrelevant tone (*ρ* = 0.72, *p* < 0.001), than the other way around (alarm on beep (*ρ* = 0.51, *p* < 0.001) irrelevant on beep (*ρ* = 0.63, *p* < 0.001). The highest correlation was found when using either the alarm or the irrelevant model to predict the neural data based on either alarm or irrelevant feature information. Despite the positive correlations, segments were always predicted better when the neural model fit the features used, compared to the cross-prediction (Table 2 and Extended Data Table 2-1, 2-2).

We also tested how models based on general acoustic properties generalize to models based on SI markers and vice versa. We observed the same positive relationship of feature models generalizing to other features. Importantly, at face values these correlational scores overall are lower compared to those of AC predicting other AC or SI other SI features (Table 2).

## Discussion

Understanding the neural response to natural soundscapes necessitates comprehensive information about the auditory environment. The abstraction of this information into features represents a fundamental parameter that influences the estimation of neural response models. Beyond depicting acoustic aspects the soundscape can also be described in terms of how the perceiver interacts with it. For this, we derived SI markers and CPs. We set out to test in how far the features’ comprehensiveness in depicting the soundscape impacts the amount of neural variability that can be explained.

Our findings show that as we provide both, SI information and a more acoustically detailed description of the auditory signal, the accuracy of model prediction improves. Simultaneously, deriving the most parsimonious abstraction of the complex soundscape, that is the acoustic onset of sounds, can be used to explain significant portions of neural variability. These findings provide crucial insights to understand and determine what aspects of the naturalistic auditory soundscape are important to capture to investigate the underlying neural activity.

### Features comprehensiveness’s impact on explaining neural variability

For the acoustic aspects, we abstracted the complex soundscape in varying degrees of acoustic detail to determine the impact on explaining neural variability. Features ranged from the most fundamental aspects, the onset of sounds, to the envelope, and the highly detailed mel-spectrogram. Replicating previous results, we find that acoustic features explain significant portions of neural data, where the explained neural variability increases with the acoustic detail of the features (Drennan and Lalor, 2019; Desai et al., 2021). Here the mel-spectrogram explains most neural variability, replicating the results of previous studies (Di Liberto et al., 2015; Daube et al., 2019). This can be explained by the feature’s characteristic resembling human auditory processing, the decomposition of the signal into non-linear spaced frequency bands, which captures the non-linear neural response more accurately (Rahman et al., 2020; Brodbeck et al., 2023). Interestingly, the most parsimonious representation, the acoustic onsets, explains a significant portion of the data, that depending on the analysis, is on par with the continuous envelope.

Close examination of combining acoustic features—onsets, with either the envelope or mel-spectrum—reveals no enhancement of prediction accuracy, suggesting they explain the similar aspects of the neural signal. This finding aligns with our variance partitioning results, indicating shared explained neural variance among these features. The information overlap is attributed to a common basis: onsets are derived from the envelope, which is in some sense a broadband representation of the mel-spectrogram.

Furthermore, the generalization between acoustic onsets and the mTRF envelope underscores their high comparability. From this, we contend that the predominant information content in the mTRF envelope relevant to deriving a neural response model is largely associated with acoustic transients. This assertion aligns with established findings (Zuk et al., 2020; Deoisres et al., 2023; Oganian et al., 2023), supporting the notion that acoustic transients are a key determinant in driving the neural response. Notably, our findings gain further significance when considering that, upon excluding samples with no predictions, the mTRF envelope and acoustic onsets are not significantly different from each other. One could argue against this line of reasoning by pointing out that peak latency differences between the acoustic onset and mTRF envelope model had to be corrected for the cross-prediction analysis. Subsequently, these peak differences could suggest separate neural processing. Here, we would like to refer to the work of Lalor et al. (2009) who contrasted the response functions to discrete unit impulses and continuous stimulus characteristics. Not only did they observe a similar shift of peak latencies for discrete and continuous stimuli, but also their source localization revealed the continuous model to be a generalized version of the discrete response. The shift of latencies intuitively makes sense given that the envelope reaches maximum energy later compared to the discrete onsets. Here, the neural response is closer to the later occurring peak of the envelope, resulting in earlier peak latencies for the continuous model (Brodbeck et al., 2018). The finding that acoustic onsets capture crucial aspects of a complex soundscape is particularly relevant as acoustic onsets represent only a fraction of the soundscape, making them a suitable feature for BTL recordings (Hölle and Bleichner, 2023).

Besides the abstraction into acoustic features, we also depicted the soundscape in terms of how the user interacts with it, using sound identity markers and CPs. Regarding SI markers, there are considerable differences in their predictive power. Specifically, the irrelevant and alarm sounds show higher prediction accuracy compared to the beep tone. This is likely due to their heightened salience (Huang and Elhilali, 2017), evoking stronger neural responses. Despite the SI models’ uniquely explained data, the cross-prediction suggests model weights to be similar. We argue that weights fitted on specific sounds share a common neural basis and that the observed variations are partly due to differences in the acoustic profiles of the sounds.

It is important to note that there is not a single “best” feature; several distinct feature sets could all yield the similar prediction accuracy. Therefore, it is crucial to keep the research question in mind and recognize that a feature set explaining a large proportion of the variance might provide a plausible explanation, but it is only one of many possible explanations for the observed data (Diedrichsen, 2020). This complexity underscores the need to consider multiple feature combinations and remain cautious about over-interpreting the significance of any single feature set. The results in this study indicate a general pattern, where the set of features that explain the most neural variability is a combination of detailed acoustic features and SI markers. The overall improvement of combining acoustic features with SI markers can be explained by SI markers accounting for sound-specific variance and acoustic models representing an average neural response to acoustic aspects. For instance, we argue that the acoustic onsets represent a suboptimal one-fits-all solution, as they encompass a broad range of different sounds, thus yielding weights representing a smeared average. Conversely, SI models, derived from recurring sounds, offer a more accurate representation of the underlying processing. The similarity between these models shown by the cross-predictions supports the notion that general acoustic processing properties are represented in both types of models. Somewhat related findings come from speech analysis, where Di Liberto et al. (2015) found that the inclusion of SI markers, in this case, phonemes, improved prediction accuracies compared to a model where only the spectrogram was used. Whether the improved prediction is solely due to a more refined estimation of the acoustic processing to the specific sounds (Daube et al., 2019), or shows higher-order processing cannot be determined from these results alone and requires further investigation.

Generally, our results suggest that general acoustic models are suitable to explain neural variability, but can be improved by accounting for sound-specific variance. However, these results should be interpreted cautiously. The sounds in our study that have identity markers are simple beep-like and presented identically each time they occur. This contrasts with the rest of the signal, where the acoustics are more varied. Despite this variability, we can reasonably hypothesize that providing more detailed information about SI can significantly improve prediction accuracy. For studies, where this information is not readily available (Hölle et al., 2022), this proves to be a vital finding, as efforts should be directed toward obtaining better descriptors of the acoustic environment when recording beyond the lab. One way to obtain a better description of the acoustic environments where sound identity information is not readily available, novel online, deep neural nets such as the Yamnet could be applied. The continuous classification of sound categories could aid in comprehensively describing the soundscape and thus improve neural model estimation.

### Comparing discrete to continuous features

Beyond exploring acoustic properties, SI, and cognitive priors, our study extends to understanding how the temporal distribution of sounds and their representation as features affect model weight estimation in EEG analysis.

An important aspect to consider when comparing continuous and discrete features is that the latter only explains specific sections of the data. To ensure an accurate assessment of model performance, we chose to concentrate on EEG data segments following auditory events and exclude “event-free” periods from the correlation computation. Here, the derived feature does not contain a depiction of the soundscape, thus no mapping onto the neural data can occur. The prediction is essentially zero. Including periods where no event was detected will impact the computation of the correlation. For the SI markers, this is most vivid as they significantly outperform acoustic features in terms of prediction accuracy. It has to be noted, however, that they apply to only a limited portion of the overall data and are repeated identically in the soundscape. Whether the observed effect holds for soundscapes without repeating identical tones has to be shown. Yet, the relative decrease of prediction accuracy for discrete features is seldom recognized in studies that contrast discrete with continuous features (Mesik and Wojtczak, 2023). Here, researchers should decide whether a comparison is suitable over samples that can be predicted, or whether the inability of discrete features to predict every sample should be factored into the comparison to continuous features.

It has to be noted that our approach to derive acoustic onsets uses a somewhat arbitrary threshold of what is considered an onset and what is ignored. This approach originated from our previous study (Hölle et al., 2022). This has several implications for the analyses. Specifically, the selection of the threshold alters the type of onsets detected and thus inevitably the derived model and hence the prediction accuracy. Although the choice of the threshold is arbitrary to some degree a too-low threshold will result in many onsets being detected, ranging from clearly audible to minor, barely perceptible sound gains. These might not be meaningful when setting the brain in relation to the acoustic description. Concurrently, a too-high threshold will limit the analysis to only very few distinct sounds, failing to describe large portions of the soundscape. To strike the balance of this selection is difficult and will depend ultimately on the choice of the researcher. Despite the arbitrary nature of threshold selection, we have shown that the most fundamental depiction of a soundscape: the onset of sounds is sufficient to explain neural variability.

This study contributes to the field by extending findings from speech studies to complex soundscapes, such as the operating room environment we used here. We demonstrate that approaches commonly used for studying speech tracking—such as breaking down the acoustic signal into different feature sets (e.g., phonemes, word surprisal)—can also be applied to natural soundscapes. Similar to speech tracking, different feature sets can capture different aspects of the soundscape.

It is important to note that the nature of the soundscape (e.g., man-made vs. natural sounds) and our relationship to these sounds can influence which feature sets are most informative. For instance, a few studies have looked at processing differences of speech and music using EEG (Zuk et al., 2020; Shan et al., 2024). Future studies could therefore explore the interaction between speech and non-speech aspects of the soundscape to further understand these dynamics.

### Conclusion

In this study, we have shown that estimating the neural response to a naturalistic soundscape is possible using a combination of acoustic features, sound-identity information, and CPs. While parsimonious acoustic onsets suffice for robust neural modeling, a detailed and specific description of the soundscape generally enhances model estimation. However, this specific information is not consistently available when relating the neural signal to an everyday soundscape. For instance, neither the cognitive state of the perceiver nor the exact sound labels may be known when monitoring everyday life sound perception.

This variability in feature availability underscores the nuanced nature of EEG data analysis in natural soundscapes. Our findings do not pinpoint a single “optimal” feature set but rather highlight several advantages and limitations to consider. By addressing these challenges, we aim to provide a more holistic understanding and set the groundwork for the future research in EEG analysis of complex auditory environments.

## Synthesis

Reviewing Editor: Anne Keitel, University of Dundee Decisions are customarily a result of the Reviewing Editor and the peer reviewers coming together and discussing their recommendations until a consensus is reached. When revisions are invited, a fact-based synthesis statement explaining their decision and outlining what is needed to prepare a revision will be listed below. The following reviewer(s) agreed to reveal their identity: Amir Chalehchaleh. Note: If this manuscript was transferred from JNeurosci and a decision was made to accept the manuscript without peer review, a brief statement to this effect will instead be what is listed below.

The two reviewers and the editor would like to thank the authors for their thorough review and the added analyses. We found the manuscript overall interesting and timely. Nonetheless, the reviewers had a few requests for adding more methodological detail, potential analyses, and for conceptual clarifications.

Please find the unabridged reviewer comments below. We ask that you respond to each comment in a point-by-point manner.

*****

Reviewer #1

Advances the Field (Required)

It effectively investigates to which level of abstraction acoustic information influence the captured neural response.

Comments to the Authors (Required)

This work effectively examines the impact of acoustic features at different level of detail on prediction accuracy and explores whether incorporating information about sound identities and their context enhances the model's predictions. However, there are a few issues that I found noteworthy:

1- The cognitive prior feature is not well described, and it could be very helpful to clearly explain how this feature set is obtained

2- looking into correlation gain in a forward selection or backward elimination step could be very helpful in determining the amount of influence each feature has in predicting the neural data

3- In addition to comparing prediction correlation, it would be beneficial to also compare the TRF weights in terms of lag difference and also amplitude

4- line 602-608; there is no such thing as a comparison figure contrasting mTRF envelope and envelope. (Editor comment: This likely referred to the initial submission that did not include the revisions, please ignore if this is the case)

*****

Reviewer #2

Advances the Field (Required)

I think the setup of this study, using a naturalistic OR soundscape, is fairly unique in TRF work. The analyses are extensive, and some innovations (looking at density of events) may be referred to in the future, but I think they contribute less conceptually than their application to the naturalistic soundscape.

Extended Data

The extended data includes the code used to analyze the data and generate the figures (if I understand it correctly)

Software Comments

Analyses were carried out in Matlab using freely available toolboxes that are widely used.

Comments to the Authors (Required)

The authors examine EEG recorded during a naturalistic soundscape, quantifying the effect of tagging individual salient events in addition to the general acoustic features. The authors have clearly put in a lot of work analyzing this data from many angles, and I appreciated their responses to reviewers (I am joining after a round of review). I have a few conceptual comments, though, that if addressed I think will improve the manuscript.

One of the takeaways appears to be "...the importance of a comprehensive description of the soundscape..." (Abstract). I think this is self-evident without context and it's underselling the results. Can you give more details? Which features had the most predictive power when characterizing your soundscape? I suggest addressing this in more detail in the abstract (and maybe the discussion in section 4.1, perhaps focusing more on the most relevant features).

Relating to this point, Reviewer #1 questioned why the speech in the natural soundscape was not analyzed, but I don't think this was sufficiently addressed by the authors. Note that the analyses applied here have been primarily used for speech, so seeing it applied to everything except the speech might raise eyebrows. I don't think more analyses are necessary, but please explain in a little more detail the reason for why speech was ignored here. This is especially important if having a "comprehensive description of the soundscape" is a main result.

Lastly, the authors' conclusion that event-free data should be ignored ("To ensure an accurate...correlation comprehension", sec. 4.2 lines 855-857) is perhaps the wrong conclusion. If you focus on sections with actual events, then prediction accuracy should go up because you are isolating segments with signal (Figure 6, also Reviewer #1's point). But for most realistic scenarios for applying this analysis (e.g. real-time prediction of EEG), understanding how the model does in event-free windows is also useful. For example, looking at event-free windows can show if the model is capturing acoustic features unrelated to, but correlated with, the original source. As an alternative, section 4.2 can maybe be reframed by elaborating on the specific contexts in which rejecting these event-free windows should be done, explaining what is gained in doing so.

Detailed comments:

- Figure 1B: The spectrogram's details are hard to see. Can you change the max value of the color plot so that some of the lower-amplitude information is clearer?

- Sec. 2.2.4, line 285: change "one's" to "ones"

- Sec. 2.4, line 304 (and maybe later): "split the data into segments" - How long (in duration) is each segment?

- Sec. 2.4.3: Please specify how many events there are in "high density" and "low density" segments - since this was based on the ranked number of events, you can do this by saying "> [??] events" for high density and "<[??] events" for low density.

- Figure 5A: Why are there stars missing for some of the comparisons? If the comparisons are not significant, please label them "N.S."

- Sec. 3.4, lines 724-725: "Interestingly...timing of the peaks." I recommend looking at (and maybe citing) Lalor et al, 2009, J Neurophysiol, who found something similar when quantifying events by their onsets vs duration (I am not an author in this paper).

- Sec. 3.5.1, lines 762-766: Do you have data and stats for this? Is this referring to some figure?

- Sec. 4.2, lines 871-872: "A simulation analysis...amplitude modulation." Didn't the authors do this simulation analysis? (Figure 13 from the reviewer comments). If so, I recommend including it as a supplemental figure.

- Figure S2: For the y-axis, did you mean "Arbitrary Units" or "A.U.", not "Amplitude"?

## Author Response

***** Reviewer #1 We would like to thank the reviewer for reading our resubmission and the valuable feedback highlighting parts of the manuscript that could be further improved. We hope to have addressed all the points of concern sufficiently.

Two of the valuable suggestions for improvement involved simulating data for the proportion explained and event density analysis to further support our findings. Given their supportive nature, we initially included these analyses in the supplement to avoid lengthening the manuscript. However, since eNeuro allows only extended data, we decided to integrate both analyses into the main text. This addition, however, further increased the length of the manuscript. To avoid overwhelming the readers and due to the particular interest in the event density analysis, we have decided to remove that analysis from the current manuscript and plan to present it in a separate paper. Here we would like to specifically thank the reviewers for their feedback, motivating further analyses which aided the idea development of covering all these different aspects in a separate paper.

Advances the Field (Required) It effectively investigates to which level of abstraction acoustic information influence the captured neural response.

Comments to the Authors (Required) This work effectively examines the impact of acoustic features at different level of detail on prediction accuracy and explores whether incorporating information about sound identities and their context enhances the model's predictions. However, there are a few issues that I found noteworthy:

1- The cognitive prior feature is not well described, and it could be very helpful to clearly explain how this feature set is obtained We thank the reviewer for this helpful feedback and agree that the cognitive prior was not sufficiently introduced. We have adapted the method section as follows: added Section 2.2.5 line 293:

Cognitive Priors Besides acoustic characteristics, the cognitive state of the perceiver critically impacts the neural response to any type of auditory stimulation. For example, a cognitive prior could be the instruction given to a participant to pay attention to a specific sound. When a participant is instructed to focus on a particular sound, the neural response is generally larger compared to when the sound is unattended (Rosenkranz et al., 2023). This information cannot be derived from acoustic properties alone but is crucial for understanding the neural response.

In the current data set, there were two conditions where participants receive separate instruction: attend to the alarm sound (narrow), or attend to the beep tone (wide). The attentional manipulation should impact the perception of the soundscape and thus the underlying neural activity. Including this information in the model estimation should lead to more neural variability being explained. Specifically, we have integrated this information by building models separately for the different conditions. Exemplary for the alarm tone, we used two feature vectors, one indicating the sound onset in the narrow condition and another vector depicting the alarm tone onsets in the wide condition.

2- looking into correlation gain in a forward selection or backward elimination step could be very helpful in determining the amount of influence each feature has in predicting the neural data We thank the reviewer for the suggestion and agree that nested model analyses are interesting in determining the amount of variability each feature explains. While we did not perform a traditional elimination procedure, we applied a variance partitioning approach in Section 3.4, where features are added successively to determine their impact on prediction performance. This approach allowed us to assess the unique and shared variance explained by each feature, to understand the feature importance. We chose variance partitioning because it provides a comprehensive view of how different features contribute to the model and is well-suited for handling the complex, interrelated nature of our data. Additionally, this method avoids potential overfitting issues that could arise with elimination procedures given our dataset's size and complexity.

3- In addition to comparing prediction correlation, it would be beneficial to also compare the TRF weights in terms of lag difference and also amplitude We agree with the reviewer that it can be informative to compare TRFs weight in terms of lag differences and amplitude. The interpretation and comparison of TRF lag and amplitudes is straightforward when the underlying feature vectors are identical and only the instruction to the participant changes (e.g. in a dual speaker paradigm in which participants are instructed to listen to one of the streams while everything else stays the same). However, we here deliberately avoid this step. In our case, we would compare TRFs that are created using different types of feature vectors. In this case, the comparison of the amplitudes and lag between feature types is not straightforward anymore. The observed differences could be due to the properties of the feature vectors. An example of this would be the comparison of onsets and envelopes, where the first negative weight peak occurs at a shorter latency for the envelope. This, however, is due to the envelope gaining maximum power later than the onsets, thus being closer to the actual neural response (Brodbeck et al., 2018). In one of the comments of Reviewer 2 an interesting source was suggested that has looked to some degree into this matter.

4- line 602-608; there is no such thing as a comparison figure contrasting mTRF envelope and envelope. (Editor comment: This likely referred to the initial submission that did not include the revisions, please ignore if this is the case) This indeed refers to the initial submission and is now being addressed in 2.2.2. ***** Reviewer #2 We would like to express our gratitude to the reviewer for thoroughly reading our manuscript and providing detailed feedback, which we believe has improved our manuscript tremendously. We hope that we were able to answer all questions and adapt the manuscript appropriately.

Previous suggestions involved simulating data for the proportion explained and event density analysis to further support our findings. As was suggested by the Reviewer #2, we included the density analysis also in the manuscript. Given their supportive nature, we initially included both analyses in the supplement to avoid lengthening the manuscript. However, since eNeuro allows only extended data, we decided to integrate both analyses into the main text. This addition, however, further increased the length of the manuscript. To avoid overwhelming the readers and due to the particular interest in the event density analysis, we have decided to remove that analysis from the current manuscript and plan to present it in a separate paper. Here we would like to specifically thank the reviewers for their feedback, motivating further analyses which aided the idea development of covering all these different aspects in a separate paper.

Advances the Field (Required) I think the setup of this study, using a naturalistic OR soundscape, is fairly unique in TRF work. The analyses are extensive, and some innovations (looking at the density of events) may be referred to in the future, but I think they contribute less conceptually than their application to the naturalistic soundscape.

Extended Data The extended data includes the code used to analyze the data and generate the figures (if I understand it correctly) Software Comments Analyses were carried out in Matlab using freely available toolboxes that are widely used.

Comments to the Authors (Required) The authors examine EEG recorded during a naturalistic soundscape, quantifying the effect of tagging individual salient events in addition to the general acoustic features. The authors have clearly put in a lot of work analyzing this data from many angles, and I appreciated their responses to reviewers (I am joining after a round of review). I have a few conceptual comments, though, that if addressed I think will improve the manuscript.

One of the takeaways appears to be "...the importance of a comprehensive description of the soundscape..." (Abstract). I think this is self-evident without context and it's underselling the results. Can you give more details? Which features had the most predictive power when characterizing your soundscape? I suggest addressing this in more detail in the abstract (and maybe the discussion in section 4.1, perhaps focusing more on the most relevant features).

We appreciate the reviewer's comment and understand the urge to have a clear-cut indication of which feature is the best one. Whereas for the acoustic features, our findings validate the results of previous studies, we shy away from making such a determination for larger feature sets, as this question cannot be generally answered. The core of our argument is that we must account for the complexity inherent in the data and the soundscape. The main motivation to not compare several sets of features is based on the work of Diedrichsen (2020) who coined the term fallacy: "over-interpreting the significance of specific feature sets constitutes an intellectual dead-end". He argues that many different feature combinations could yield the same prediction accuracy, thus "debates about which feature set is most appropriate miss the point". It appears that we did not make this point sufficiently clear in our original manuscript, so we have now made it more explicit in the abstract and throughout the discussion.

Abstract line 13 "The results demonstrated that the feature sets that explain the most neural variability were a combination of highly detailed acoustic features with a comprehensive description of specific sound onsets." We have added a small section discussing the best-performing acoustic feature in Section 4.1 Line 687 Here the mel-spectrogram explains most neural variability, replicating the results of previous studies (Daube et al., 2019; Di Liberto et al., 2015). This can be explained by the feature's characteristic resembling human auditory processing, the decomposition of the signal into non-linear spaced frequency bands, which captures the non-linear neural response more accurately (Brodbeck et al., 2023; Rahman et al., 2020).

We have included this reasoning in the discussion to make this point clearer to the reader Section 4.1 line 731.

It is important to note that there is not a single "best" feature; several distinct feature sets could all yield similar prediction accuracy. Therefore, it is crucial to keep the research question in mind and recognize that a feature set explaining a large proportion of the variance might provide a plausible explanation, but it is only one of many possible explanations for the observed data (Diedrichsen, 2020). This complexity underscores the need to consider multiple feature combinations and remain cautious about over-interpreting the significance of any single feature set. The results in this study indicate a general pattern, where the set of features that explain the most neural variability is a combination of detailed acoustic features and sound identity markers. The overall improvement of combining acoustic features with sound identity markers can be explained by sound identity markers accounting for sound-specific variance and acoustic models representing an average neural response to acoustic aspects. For instance, we argue that the acoustic onsets ...." Relating to this point, Reviewer #1 questioned why the speech in the natural soundscape was not analyzed, but I don't think this was sufficiently addressed by the authors. Note that the analyses applied here have been primarily used for speech, so seeing it applied to everything except the speech might raise eyebrows. I don't think more analyses are necessary, but please explain in a little more detail the reason for why speech was ignored here. This is especially important if having a "comprehensive description of the soundscape" is a main result.

We appreciate the reviewer's comment regarding the omission of the speech stimuli. We deliberately decided against analyzing the speech material for the following reasons:

1. Our primary aim is to investigate complex acoustic environments where speech is just one element and could also be entirely absent. Including speech, analysis would shift the focus of this already comprehensive paper toward a stimulus that has been extensively studied in isolation. Our intention was to explore how non-speech elements, which are less frequently examined, contribute to neural responses in naturalistic settings.

2. By focusing on non-speech sounds, we aim to extend the application of features traditionally used in speech studies to broader and more varied soundscapes. This allows us to determine whether these features are suitable for modeling complex auditory environments beyond the speech domain.

We have added this rationale to the manuscript to provide greater clarity (Section 2.2, line 191).

These features have been validated in many different studies using speech stimuli (Brodbeck et al., 2018; Daube et al., 2019; de Heer et al., 2017; Desai et al., 2021; Mesik &Wojtczak, 2023). Here we want to test whether these features can be extended to soundscapes with non-speech soundscapes.

Additionally, in Section 4.2 (line 801), we highlight the potential for future research. "This study contributes to the field by extending findings from speech studies to complex soundscapes, such as the operating room environment we used here. We demonstrate that approaches commonly used for studying speech tracking-such as breaking down the acoustic signal into different feature sets (e.g., phonemes, word surprisal)-can also be applied to natural soundscapes. Similar to speech tracking, different feature sets can capture different aspects of the soundscape.

It is important to note that the nature of the soundscape (e.g., man-made vs. natural sounds) and our relationship to these sounds can influence which feature sets are most informative. For instance, a few studies have looked at processing differences of speech and music using EEG (Shan et al., 2024; Zuk et al., 2020). Future studies could therefore explore the interaction between speech and non-speech aspects of the soundscape to further understand these dynamics.

Lastly, the authors' conclusion that event-free data should be ignored ("To ensure an accurate...correlation comprehension", sec. 4.2 lines 855-857) is perhaps the wrong conclusion. If you focus on sections with actual events, then prediction accuracy should go up because you are isolating segments with signal (Figure 6, also Reviewer #1's point). But for most realistic scenarios for applying this analysis (e.g. real-time prediction of EEG), understanding how the model does in event-free windows is also useful. For example, looking at event-free windows can show if the model is capturing acoustic features unrelated to, but correlated with, the original source. As an alternative, section 4.2 can maybe be reframed by elaborating on the specific contexts in which rejecting these event-free windows should be done, explaining what is gained in doing so.

We are not entirely sure if we understand the comment correctly. It may be helpful to clarify what we consider as "event-free" segments. Specifically, we refer to moments where there is no sound depicted in the feature. In these "event-free" periods, the prediction model does not generate a meaningful prediction (i.e., it predicts a null response). When this null prediction is compared with the EEG data from those periods, it will not correlate, as there is no acoustic input to drive the neural response.

Including these segments when reconstructing the signal would trivially reduce the correlation, as the model is not designed to account for non-acoustic neural activity. Therefore, we argue that it is not meaningful to include these "event-free" segments in our analysis, as they do not contribute to understanding the model's predictive performance regarding sound-driven neural activity. We now make this more explicit in the text.

Line 775 "Here the derived feature does not contain a depiction of the soundscape, thus no mapping onto the neural data can occur. The prediction is essentially zero. Including periods where no event was detected will impact the computation of the correlation." - Figure 1B: The spectrogram's details are hard to see. Can you change the max value of the color plot so that some of the lower-amplitude information is clearer? We have adjusted the plot according to the recommendation of the reviewer which has improved to visibility of lower amplitude information.

- Sec. 2.2.4, line 285: change "one's" to "ones" The line has been changed.

- Sec. 2.4, line 304 (and maybe later): "split the data into segments" - How long (in duration) is each segment? We have added the information of the rough duration of each segment to the manuscript. Since the measurements between conditions and participants were not of equal length, the segment length differs. Line 348 6 segments (M =3.14, SD = 0.18 min.), - Sec. 2.4.3: Please specify how many events there are in "high density" and "low density" segments - since this was based on the ranked number of events, you can do this by saying "> [??] events" for high density and "<[??] events" for low density.

We thank the reviewer for the comment as the descriptive statistics of the density analysis allows the reader to gain an intuition of the number of events between high and low segments. Given the continued interest and the already lengthy manuscript we have decided to remove the analysis completely and cover it in more detail in a separate paper.

- Figure 5A: Why are there stars missing for some of the comparisons? If the comparisons are not significant, please label them "N.S." We thank the reviewer for thoroughly reading the figures. Indeed, missing stars were used to symbolize the non-significance of the comparisons. We have adjusted figures 3 and 4 to keep them consistent with this suggestion of the reviewer as well as the descriptions of the corresponding figures.

- Sec. 3.4, lines 724-725: "Interestingly...timing of the peaks." I recommend looking at (and maybe citing) Lalor et al, 2009, J Neurophysiol, who found something similar when quantifying events by their onsets vs duration (I am not an author in this paper).

We thank the reviewer for the suggested paper, which the authors were not aware of. One important aspect this paper highlights is that TRF (AESPA) functions take stimulus properties into account, which modulate the estimated responses. We have added this source in our discussion as follows line 708:

One could argue against this line of reasoning by pointing out that peak latency differences between the acoustic onset and mTRF envelope model had to be corrected for the cross-prediction analysis. Subsequently, these peak differences could suggest separate neural processing. Here we would like to refer to the work of (Lalor et al., 2009) who contrasted the response functions to discrete unit impulses and continuous stimulus characteristics. Not only did they observe a similar shift of peak latencies for discrete and continuous stimuli, but also their source localization revealed the continuous model to be a generalized version of the discrete response. The shift of latencies intuitively makes sense given that the envelope reaches maximum energy later compared to the discrete onsets. Here, the neural response is closer to the later occurring peak of the envelope, resulting in earlier peak latencies for the continuous model (Brodbeck et al., 2018). The finding that acoustic onsets capture crucial aspects of a complex soundscape is particularly relevant as acoustic onsets represent only a fraction of the soundscape.

- Sec. 3.5.1, lines 762-766: Do you have data and stats for this? Is this referring to some figure? We again thank the reviewer for the thorough read. The data that we are referring to is presented in Table 1 which is not referenced in the manuscript. We have adapted this. Line 663s Other SI features (Table 1).

- Sec. 4.2, lines 871-872: "A simulation analysis...amplitude modulation." Didn't the authors do this simulation analysis? (Figure 13 from the reviewer comments). If so, I recommend including it as a supplemental figure.

We did indeed perform a simulation analysis. We did not include it so far, as said during the previous round of revisions, we were unsure whether this analysis would be better suited in a paper of its own. Given the continued interest and the already lengthy manuscript we have decided to remove the analysis completely and give more detailed attention in a separate paper.

- Figure S2: For the y-axis, did you mean "Arbitrary Units" or "A.U.", not "Amplitude"? We again thank the reviewer for the thorough read and excuse this mistake. The axis label should indeed be arbitrary units instead of amplitude. We have changed this.

- References Brodbeck, C., Das, P., Gillis, M., Kulasingham, J. P., Bhattasali, S., Gaston, P., Resnik, P., &Simon, J. Z. (2023). Eelbrain, a Python toolkit for time-continuous analysis with temporal response functions. eLife, 12, e85012. https://doi.org/10.7554/eLife.85012 Brodbeck, C., Hong, L. E., &Simon, J. Z. (2018). Rapid Transformation from Auditory to Linguistic Representations of Continuous Speech. Current Biology: CB, 28(24), 3976-3983.e5. https://doi.org/10.1016/j.cub.2018.10.042 Daube, C., Ince, R. A. A., &Gross, J. (2019). Simple Acoustic Features Can Explain Phoneme-Based Predictions of Cortical Responses to Speech. Current Biology, 29(12), 1924-1937.e9. https://doi.org/10.1016/j.cub.2019.04.067 de Heer, W. A., Huth, A. G., Griffiths, T. L., Gallant, J. L., &Theunissen, F. E. (2017). The Hierarchical Cortical Organization of Human Speech Processing. The Journal of Neuroscience, 37(27), 6539-6557. https://doi.org/10.1523/JNEUROSCI.3267-16.2017 Desai, M., Holder, J., Villarreal, C., Clark, N., Hoang, B., &Hamilton, L. S. (2021). Generalizable EEG Encoding Models with Naturalistic Audiovisual Stimuli. The Journal of Neuroscience, 41(43), 8946-8962. https://doi.org/10.1523/JNEUROSCI.2891-20.2021 Di Liberto, G. M., O'Sullivan, J. A., &Lalor, E. C. (2015). Low-Frequency Cortical Entrainment to Speech Reflects Phoneme-Level Processing. Current Biology, 25(19), 2457-2465. https://doi.org/10.1016/j.cub.2015.08.030 Diedrichsen, J. (2020). Representational Models and the Feature Fallacy. In D. Poeppel, G. R. Mangun, &M. S. Gazzaniga (Hrsg.), The Cognitive Neurosciences (6. Aufl., S. 669-678). The MIT Press. https://doi.org/10.7551/mitpress/11442.003.0074 Lalor, E. C., Power, A. J., Reilly, R. B., &Foxe, J. J. (2009). Resolving Precise Temporal Processing Properties of the Auditory System Using Continuous Stimuli. Journal of Neurophysiology, 102(1), 349-359. https://doi.org/10.1152/jn.90896.2008 Mesik, J., &Wojtczak, M. (2023). The effects of data quantity on performance of temporal response function analyses of natural speech processing. Frontiers in Neuroscience, 16. https://www.frontiersin.org/articles/10.3389/fnins.2022.963629 Rahman, M., Willmore, B. D. B., King, A. J., &Harper, N. S. (2020). Simple transformations capture auditory input to cortex. Proceedings of the National Academy of Sciences, 117(45), 28442-28451. https://doi.org/10.1073/pnas.1922033117 Rosenkranz, M., Cetin, T., Uslar, V. N., &Bleichner, M. G. (2023). Investigating the attentional focus to workplace-related soundscapes in a complex audio-visual-motor task using EEG. Frontiers in Neuroergonomics, 3. https://www.frontiersin.org/articles/10.3389/fnrgo.2022.1062227 Shan, T., Cappelloni, M. S., &Maddox, R. K. (2024). Subcortical responses to music and speech are alike while cortical responses diverge. Scientific Reports, 14(1), Article 1. https://doi.org/10.1038/s41598-023-50438-0 Wang, A. L., Mouraux, A., Liang, M., &Iannetti, G. D. (2008). The Enhancement of the N1 Wave Elicited by Sensory Stimuli Presented at Very Short Inter-Stimulus Intervals Is a General Feature across Sensory Systems. PLOS ONE, 3(12), e3929. https://doi.org/10.1371/journal.pone.0003929 Woldorff, M. G. (1993). Distortion of ERP averages due to overlap from temporally adjacent ERPs: Analysis and correction. Psychophysiology, 30(1), 98-119. https://doi.org/10.1111/j.1469-8986.1993.tb03209.x Zuk, N. J., Teoh, E. S., &Lalor, E. C. (2020). EEG-based classification of natural sounds reveals specialized responses to speech and music. NeuroImage, 210, 116558. https://doi.org/10.1016/j.neuroimage.2020.116558
