# Supplementary figures and images for: Exploring Relevant Features for EEG-Based Investigation of Sound Perception in Naturalistic Soundscapes
Source: eNeuro. 2025 Jan 16;12(1):ENEURO.0287-24.2024. doi: 10.1523/ENEURO.0287-24.2024 (PMC11747973; doi:10.1523/ENEURO.0287-24.2024)

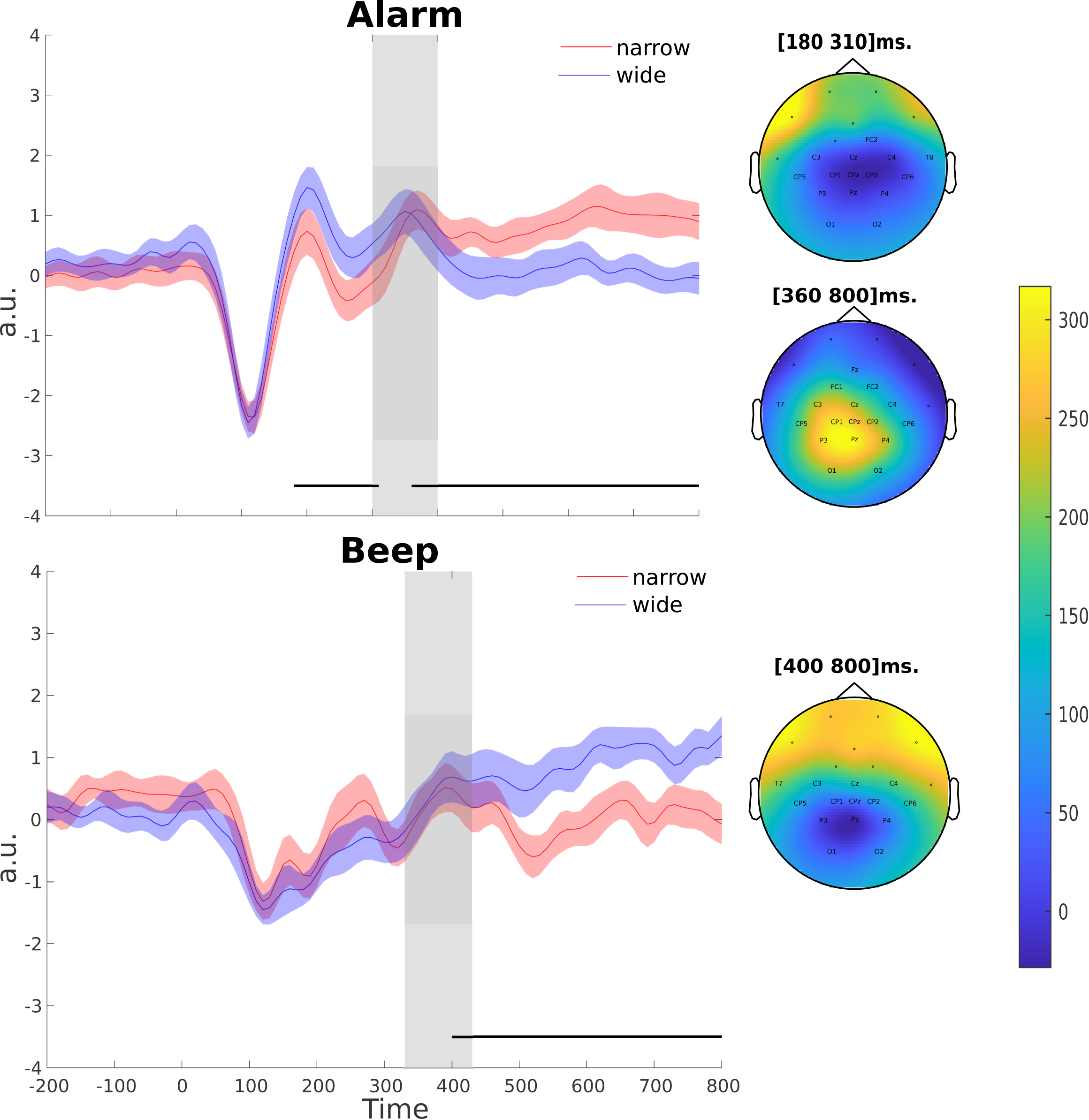

Supplement: Figure 3-1 — Upper panel shows the TRF weights of the alarm tone for the two conditions. The grey shaded area marks the window of interest as reported by Rosenkranz et al. (2023). The black lines are the clusters detected by the permutation testing with the corresponding topographies. The lower panel shows the same but for the beep tone. Download Figure 3-1, TIF file. [file eneuro-12-ENEURO.0287-24.2024-s002.tif]

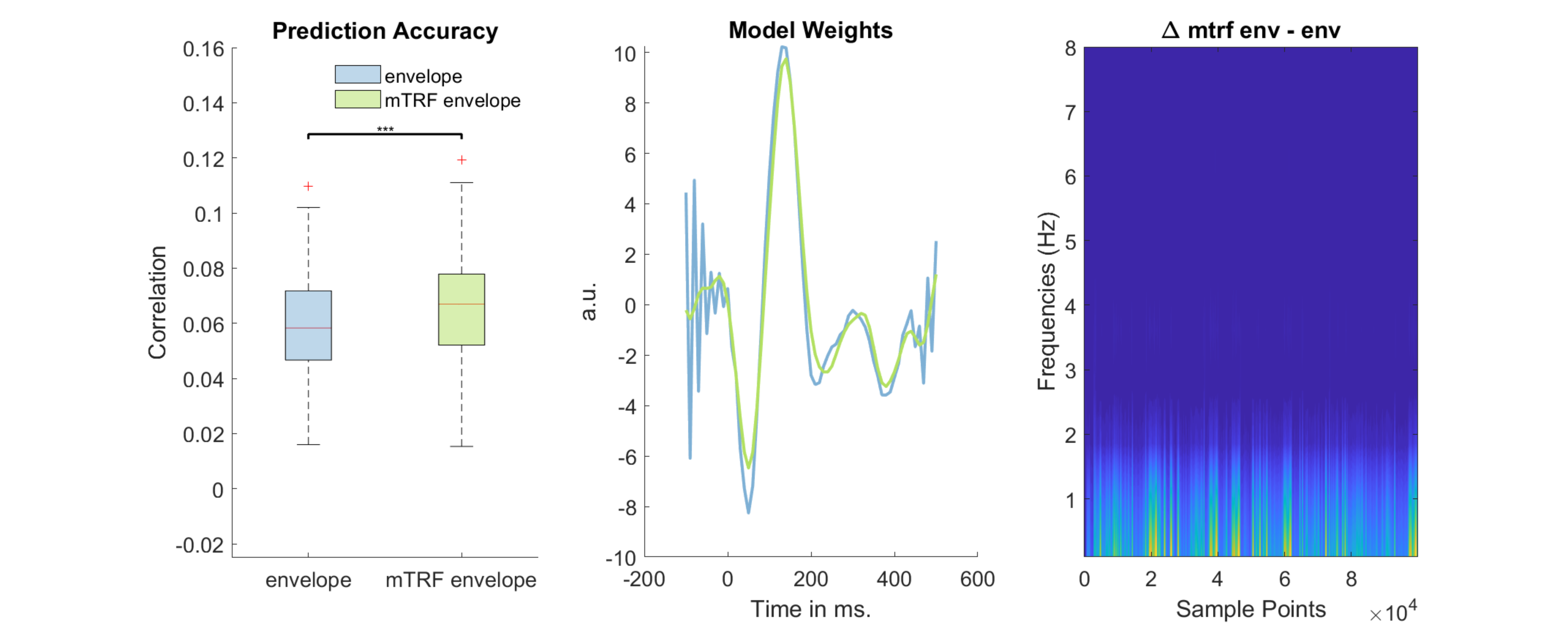

Supplement: Figure 4-1 — The left panel shows the distribution of the prediction accuracy for the envelope and mTRF envelope model. Significance is tested at * p < 0.05, **p < 0.01, ***p < 0.000. The panel in the middle shows the model weights averaged over participants, conditions, and channels. The panel on the right highlights the frequency decomposition of the difference curve of the mTRF envelope and the envelope. Download Figure 4-1, TIF file. [file eneuro-12-ENEURO.0287-24.2024-s001.tif]

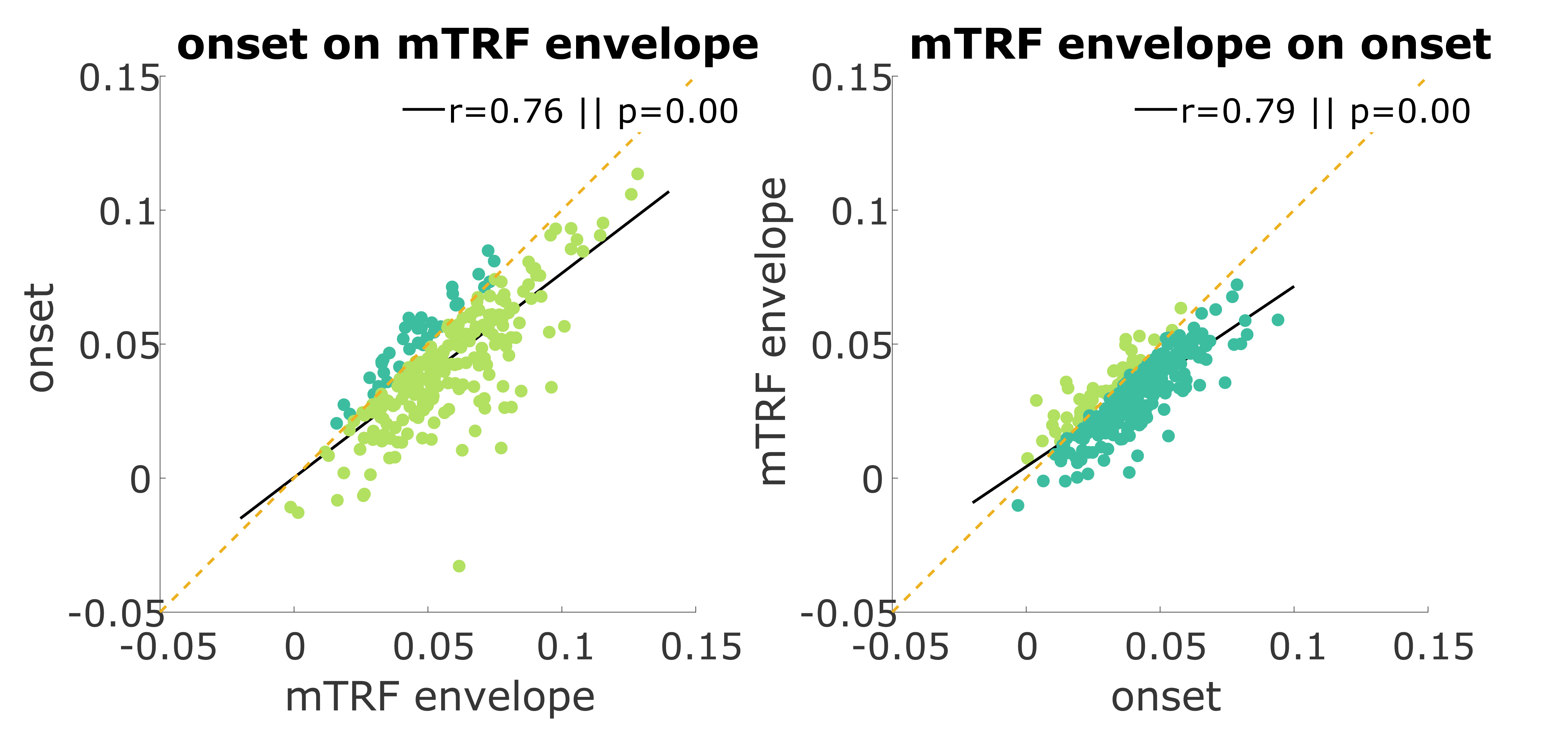

Supplement: Table 2-1 — This figure shows the results of the cross-prediction analysis. On the x-axis are the correlational score of the testing data segment with the prediction based on feature information that the model was initially trained on. On the y-axis are the correlational scores for the same segment and feature information as on the x-axis, but using model weights derived from the depicted feature. Download Table 2-1, TIF file. [file eneuro-12-ENEURO.0287-24.2024-s003.tif]

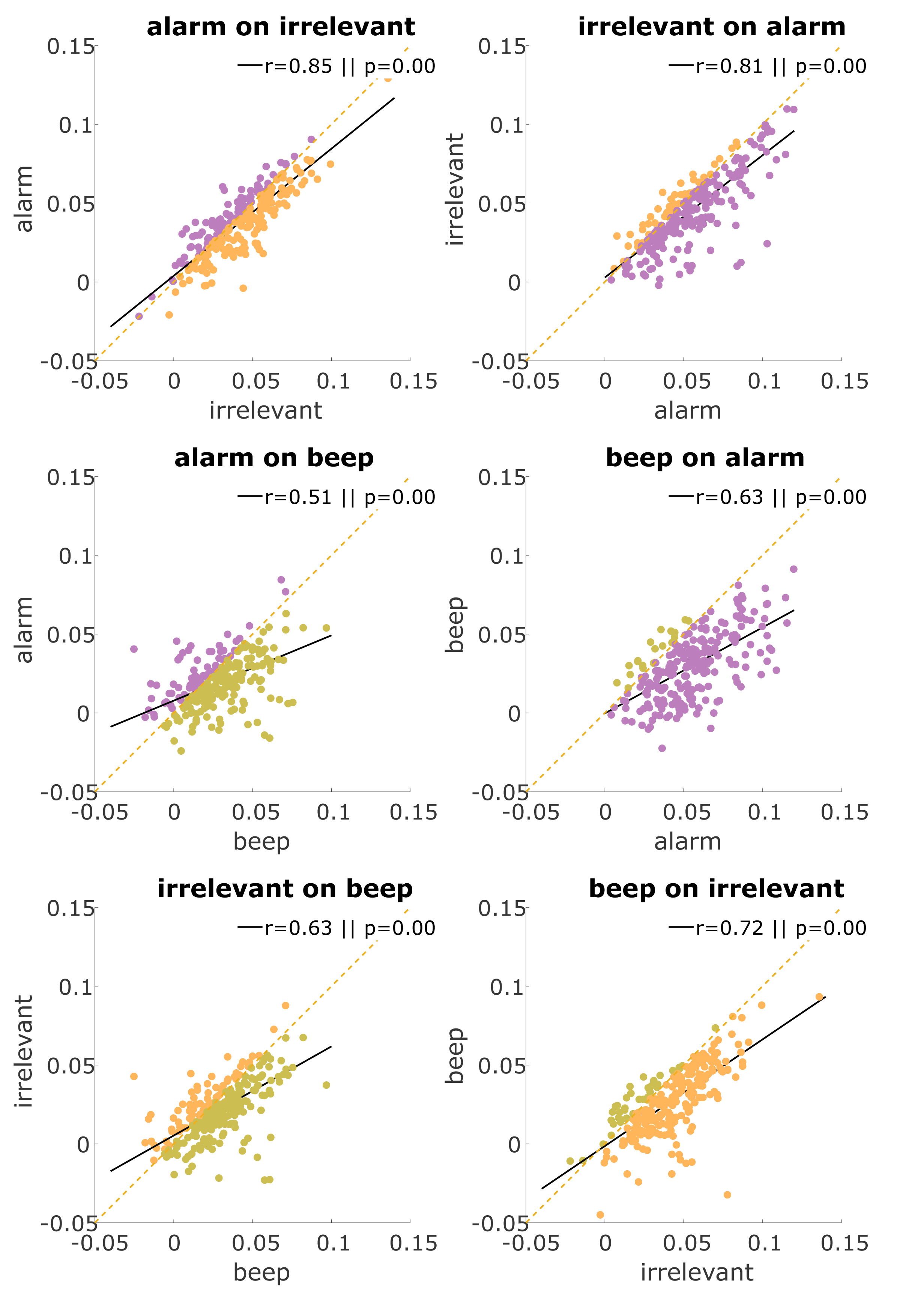

Supplement: Table 2-2 — This figure shows the results of the cross-prediction analysis for the sound identity marker. On the x-axis are the correlational scores of the testing data segment with the prediction based on feature information that the model was initially trained on. On the y-axis are the correlational scores for the same segment and feature information as on the x-axis, but using model weights derived from the depicted feature. Download Table 2-2, TIF file. [file eneuro-12-ENEURO.0287-24.2024-s004.tif]
